# Methylation-induced silencing of AZGP1 enhances prostate cancer metastasis by stimulating tumoral glycolysis

**DOI:** 10.1186/s11658-025-00818-3

**Published:** 2026-01-14

**Authors:** Lu Li, Jinguang Luo, Linyue Zhao, Lu Tian, Jianfeng Wang, Yifei Cheng, Xiao Li

**Affiliations:** 1 Department of Pathology, Nanjing Drum Tower Hospital, Affliated Hospital of Medical School, Nanjing University, Nanjing, 210008 China; 2 Department of Urology, First Affiliated Hospital of Bengbu Medical University, Bengbu, Anhui China; 3 State Key Laboratory of Quality Research in Chinese Medicine, School of Pharmacy, Macau University of Science and Technology, Macau, 999078 China; 4 Department of Urology, China–Japan Friendship Hospital, Beijing, 100029 China; 5 Department of Urology, Southeast University Zhongda Hospital, Nanjing, 210009 China; 6 Department of Urologic Surgery, Jiangsu Cancer Hospital and Jiangsu Institute of Cancer Research and Affiliated Cancer Hospital of Nanjing Medical University, Nanjing, 210009 China; 7 Department of Scientific Research, Jiangsu Cancer Hospital and Jiangsu Institute of Cancer Research and Affiliated Cancer Hospital of Nanjing Medical University, Nanjing, Jiangsu, China

**Keywords:** Prostate cancer, Metastasis, *AZGP1*, Glycolysis

## Abstract

**Background:**

Metastasis is the primary cause of mortality in patients with prostate cancer (PCa), yet effective treatments remain scarce. Identifying reliable biomarkers and understanding their underlying mechanisms is crucial for advancing clinical management.

**Methods:**

Firstly, we integrated single-cell and bulk transcriptomic data and employed the *Scissor* tool to characterize tumor cells with metastatic advantages (termed metastatic cells). Then, independent predictive genes for metastasis were identified through univariate and multivariate regression analyses. The role of hub genes in PCa metastasis was further validated using multiple large datasets, malignant phenotype experiments, in vivo metastatic models, and a clinical-sample-based immunohistochemical cohort. Further, we explored the metabolic characteristics related to hub genes through unbiased functional annotation, and validated the upregulated glycolysis by measuring l-lactic acid production, extracellular acidification rates (ECAR), and oxygen consumption rates (OCR). Finally, multi-omics data were employed to investigate the promoter-methylation-dependent regulation of alpha-2-glycoprotein 1 (*AZGP1*) transcription, with methylation confirmed through PCa cell-based methylation-specific PCR (MSP) assays.

**Results:**

*AZGP1* was identified as an independent protective predictor of metastasis, which was validated in vitro and in vivo. Metabolic functional annotation revealed that glycolysis was upregulated in *AZGP1*-positive luminal cells. Consistently, overexpression of *AZGP1* in PCa cells was associated with lower l-lactic acid levels, reduced ECAR, and increased OCR. In addition, DNA methylation at the cg26429636 region was linked to decreased transcriptional expression of *AZGP1*. MSP assays revealed an unmethylated pattern in PCa cells with high *AZGP1* expression, and higher methylation levels in *AZGP1*-low cells.

**Conclusions:**

Promoter methylation of *AZGP1* leads to reduced transcriptional expression, thereby promoting glycolysis in tumor cells and facilitating metastasis. The detection of *AZGP1* methylation levels offers a valuable reference for dynamic surveillance of PCa metastasis.

**Supplementary Information:**

The online version contains supplementary material available at 10.1186/s11658-025-00818-3.

## Introduction

Prostate cancer (PCa) is a prevalent malignancy, ranking as the second most common cancer and the fifth leading cause of cancer-related death among men worldwide [1]. Factors such as an aging population and widespread environmental pollution are contributing to a rapid increase in both the incidence and mortality of PCa in China [2]. Although the prognosis for early-stage PCa is relatively favorable, some patients experience rapid tumor progression, leading to metastasis to lymph nodes, bones, and other organs, which significantly worsens the prognosis and increases resistance to androgen deprivation therapy [3]. Currently, aside from imaging and nuclear medicine techniques, there is a lack of reliable noninvasive methods for detecting locally lymph node or distant metastases. The necessity of lymph node dissection during radical prostatectomy remains a clinical challenge. Moreover, the lack of effective therapies for metastatic disease is one of the main reasons for the poor prognosis of patients in advanced stages.

Several models have been proposed to explain the metastasis of PCa. The prevailing viewpoint suggests that tumor cells play a crucial role in the metastatic process by undergoing phenotypic changes, including decreased adhesion to surrounding cells and enhanced migratory and invasive capabilities [4]. Increasing evidence also highlights the significant role of the tumor microenvironment (TME) in tumor progression and metastasis. Primary tumors can induce the formation of a pre-metastatic TME in specific organ tissues before actual metastasis, which requires tumor-secreted factors, the recruitment of suppressive immune cells, and interactions with stromal components, such as the inflammatory polarization of the tissue [1, 2]. In addition, hypoxia and extracellular matrix remodeling further contribute to the development of the pre-metastatic TME [5]. Valastyan et al. proposed that the phenomenon of metastasis is the ultimate product of a multistep cellular biological process involving an invasion–metastasis cascade. This process encompasses the in situ infiltration and invasion of tumor cells, dissemination to distant organs, and the establishment of a conducive microenvironment for growth and proliferation by these distant organs for incoming tumor cells. Each of these events is collaboratively driven by both tumor cells and nontumor stromal cells, collectively endowing early-stage metastatic cells with the requisite features for the formation of macroscopic metastasis [3].

Moreover, metabolic reprogramming has garnered significant attention regarding its role in the reconstruction of the TME. Tumor cells utilize various metabolic pathways to meet the heightened demands for bioenergetics and biosynthesis, while simultaneously mitigating oxidative stress, which is essential for tumor cell proliferation and survival [2]. In preclinical studies, numerous metabolic molecules have been identified as potential targets for anticancer therapy [3]. During tumor metastasis, significant alterations in metabolic levels occur, and dynamic changes in the metabolism of the microenvironment, including lactate, pyruvate, glutamine, and fatty acids, can promote tumor metastasis by modifying the microenvironment of the metastatic cascade. Collectively, specific metabolic defects in cancer cells during metastasis have potential value, which can be targeted to inhibit metastatic growth and even prevent tumor colonization [4].

In this study, we conducted an in-depth analysis of tumor heterogeneity and metabolic reprogramming by integrating single-cell and bulk transcriptomic data. We unbiasedly identified *AZGP1* as a hub molecule in metastatic PCa, experimentally validated its functions, and explored its potential in liquid biopsies. We believe that our research could provide insights into new dynamic surveillance and the treatment strategies for PCa metastasis.

## Materials and methods

### Public data download and processing

The Cancer Genome Atlas Prostate Adenocarcinoma Collection (TCGA-PRAD) transcriptome data, obtained from the Genomic Data Commons data portal (GDC, https://portal.gdc.cancer.gov), contained 495 tumor samples. The mRNA expression data from TCGA-PRAD were transformed to values in transcripts per million (TPM), along with the clinical information. The samples were divided into metastatic and primary groups on the basis of the M and N grading through clinical phenotypes. Both lymph node metastasis (LM; N1 stage) and distant metastasis (M1 stage) samples were classified as metastasis groups.

GSE141445, including 36,424 single-cells from 13 prostate tumors, was used as the discovery cohort. Another single-cell dataset from Tuong et al., downloaded from https://www.omicsdi.org/dataset/ega/EGAS00001005787, included 10,587 cells and was used as validation [4]. GSE21034, DKFZ2018, and prad_su2c_2015 datasets, which contain 218, 118, and 150 PCa tumor samples, respectively, were used as independent validation cohorts.

### Evaluation of tumor metastatic ability

Markers related to tumor metastasis were collected from previous studies, and subjected to single-sample gene set enrichment analysis (ssGSEA) to calculate signature scores for the evaluation of metastatic ability. The following genes were used to construct the metastasis related gene set: *SNAI1*, *SNAI2*, *TWIST1*, *ZEB1*, and *ZEB2* for local invasion; *THBS1*, *HOMOX1*, *ITGAV*, *VHL*, *EDN1*, *JAG1*, *SMAD7*, *COL5A1*, and *COL4A1* for angiogenesis; *EDN1*, *JAG1*, *PODXL*, *TPM1*, *HMOX1*, *FURIN*, *LAMB1*, *RBPJ*, *THBS1*, and *SDAD7* for cell migration; and *TGFB1*, *CSF1*, *EGF*, *VEGFA*, *PTGS2*, *EREG*, *ANGPT2*, *MMP1*, *MMP2*, *MMP3*, and *MMP10* for extravasation [3].

### Single-cell transcriptome data preprocessing and interpreting

We applied Seurat R package (version 4.3.1) to preprocess the single-cell RNA sequencing (scRNA-seq) data. Then we collected markers for various cell types of PCa, and used *SingleR* (version 1.2.4) to annotate single cells. The cells were divided into luminal epithelial cells (*CDH1* and *AR*), basal epithelial cells (*CDH1* and *KRT5*), T cells (*CD3E*), B cells (*CD19*), fibroblasts (*ACTA2*), mast cells (*KIT*), monolytic cells (*CD14*), and endothelial cells (*CD31*), according to the annotation of Chen et al. in their original interpretation of this data. The cluster-specific marker genes were identified through *FindAllMarkers* function to verify the accuracy of cell type annotation.

### Identification of a metastatic tumor cell subpopulation of PCa

The *Scissor* tool leverages bulk data and phenotypic information from corresponding samples to identify phenotype-related cell subpopulations from single-cell data [5]. Briefly, the *Scissor* R package was used for a series of processing. Firstly, quantile normalization was applied to single-cell and batch expression data to eliminate the underlying batch processing effects. Subsequently, Pearson correlation matrices were calculated for each cell and bulk samples to quantify the similarity between single cells and bulk data. On the basis of the correlation between single-cell and corresponding batch data, selected cells were assigned as “Scissor positive” (Scissor+) or “Scissor negative” (Scissor−) cells, which are positively and negatively correlated with the phenotype of interest, respectively. By assigning samples with N1 or M1 stage as ones with a metastatic phenotype, the *Scissor* method was utilized to correlate such phenotypes with single-cell transcriptome data, categorizing luminal cells into a metastatic (Met) and a non-metastatic (non-Met) sub-population.

### Establishment of PCa metastasis model and hub gene screening

The metastasis-related genes (MRGs) were identified as those that are overexpressed in both the Met subpopulation from the discovery cohort and in samples exhibiting a metastatic phenotype from TCGA-PRAD. Correlations between MRGs and the aforementioned signatures related to metastatic ability, including local invasion, angiogenesis, cell migration, and extravasation, were analyzed to assess the relationship between MRGs and metastatic potential. Subsequently, a multivariate logistic regression model was established to measure the association between MRGs and the metastatic phenotype in TCGA-PRAD and identify independent risk MRGs. Collinearity diagnostics were initially applied to all MRGs, utilizing a variance inflation factor (VIF) to diagnose multicollinearity among them. Model construction proceeded through a stepwise forward approach using maximum likelihood estimation. The model’s score was correlated with tumor purity, immune infiltration levels, and the relationships between tumor proliferation, invasion, migration capabilities, and metabolic capacity, thereby validating findings from single-cell transcriptome analysis. Hub genes *CXCR4* and *AZGP1* were identified on the basis of regression coefficients.

Further literature review revealed AZGP1 as an established metastasis biomarker in other cancer types, yet its specific mechanisms and clinical significance in PCa metastasis remain unclear. Subsequently, sample stratification into high and low *AZGP1* expression groups was performed, followed by pathway enrichment analysis to elucidate potential biological mechanisms.

### The metabolic correlations of the Met subpopulation and AZGP1

We evaluated the activation levels of 85 metabolic pathways in KEGG using the R package *scMetabolism* for each luminal epithelial cell from the discovery cohort, with comparisons between *AZGP1*-positive (*AZGP1* expression > 0) and *AZGP1*-negative (*AZGP1* expression = 0) luminal epithelial cells, measured using the *limma* package. Then, we calculated the ssGSEA score of the glycolysis pathway in the TCGA-PRAD dataset and analyzed its correlation with *AZGP1* expression. We also analyzed the correlation between *AZGP1* expression and glycolytic enzymes, including *PFKFB3*, *SLC2A1*, *LDHA*, *PDK1*, *PGK1*, and *GP1*.

### Identification of *AZGP1* methylation regulation

On the basis of TCGA methylation and mRNA data, we assessed the correlation between *AZGP1* mRNA expression levels and the corresponding methylation levels of *AZGP1* probes. In addition, we examined the relationship between the expression levels of methylation-associated enzymes and *AZGP1*. We filtered probes located in the promoter region that exhibited a significant negative correlation with *AZGP1* expression level and a significant positive correlation with methylation-associated enzymes. Furthermore, we compared the methylation levels of these probes between M0 and M1, as well as between N0 and N1.

### Cell culture and treatments

The cell lines used in the experiment included the PCa cell lines PC3, DU145, LNCaP, 22RV1, and RM-1, which were obtained from the American Type Culture Collection (ATCC). Cells were grown in culture medium with 10% fetal bovine serum and 1% penicillin and streptomycin, and incubated in a cell culture incubator at 37 ℃ and 5% CO_2_. The functional experiments were conducted using cells in logarithmic growth phase.

In order to upregulate the expression of *AZGP1* in cells, the open reading frame (ORF) fragment of *AZGP1* (894 bp, NM_001185) was cloned into the pCMV-3 × FLAG Neo vector by Wuhan Miaoling Biotechnology Co., Ltd., and the pCMV-*AZGP1* (human) -3 × FLAG Neo plasmid was obtained.

The small interfering RNA and negative control RNA (si-NC) of *AZGP1* were designed and synthesized by Beijing Qingke Biotechnology based on the *AZGP1* sequence. The transfection efficiency was validated by reverse-transcription quantitative polymerase chain reaction (RT-qPCR).

### RNA isolation and RT-qPCR

Firstly, RNA extraction and RT-qPCR were performed. TRIzol was used according to the manufacturer’s instructions to lyse cells or tissues and extract RNA. The purity and concentration of RNA samples were further detected using an enzyme-linked immunosorbent assay (ELISA) reader. Samples were stored at −80 ℃ for later use. The RNA reverse transcription was performed using a reverse transcription kit with cDNA as a template and U6 as endogenous controls. The reaction conditions were: pre-denaturation at 95 ℃ for 10 min, 15 s at 95 ℃, and 60 s at 60 ℃, repeated for a total of 40 cycles. Finally, the relative expression level of RNA was calculated using the 2^−ΔΔCT^ method.

### Transwell migration and invasion assays

Transwell migration and invasion assays were carried out to assess the migration and invasion abilities of cells using Transwell chambers (8.0 μm pore size; Corning® BioCoat™ Matrigel). Transwell chambers coated with Matrigel (Cultrex Basement Membrane Extract, PathClear) were used for the invasion assay. A total of 200 μL of FBS-free 22RV1-1640 PC3-F12K containing 5 × 10^4^ transfected cells was placed into the upper compartment of the Transwell chambers, and the same medium supplemented with 20% serum was added to the lower chamber of each well as a chemo-attractant solution. Then, 72 h later (22RV1) and 48 h later (PC3), the remaining cells on the upper side of the membrane were wiped with a cotton swab. Cells underwent formaldehyde fixation for 15 min before PBS washing and crystal violet staining for 15 min. Under an inverted microscope, five randomly selected fields of view from each chamber were selected, allowing a count of migrating and invading cells.

### Western blotting (WB)

Total proteins were extracted and subsequently separated by 8–12% sodium dodecyl sulfate-polyacrylamide gel electrophoresis (SDS-PAGE) gels. Western blot analyses were performed according to standard procedures. The western blot loading control was β-actin (Abclonal, AC026; 1:10,000). Western blotting was performed using antibodies as follows: anti-AZGP1(Proteintech, 13399-1-AP; 1:1000), anti-MMP7 (Proteintech, 10374-2-AP; 1:1000), anti-VEGFA (Proteintech, 66828-1-Ig; 1:2000), anti-E-cadherin (Proteintech, 20874-1-AP; 1:1000), and anti-N-cadherin (Proteintech, 22018-1-AP; 1:2000).

### Wound healing assay

Cell migration was evaluated by a scratch wound healing assay. Briefly, the PRAD cancer cells were seeded on a 6-well plate at a density of 1 × 10^5^ cells/well. A scratch was made in a straight line with a 200-µl pipette tip in a confluent cell monolayer. After wounding, the monolayer was immediately cleaned and incubated with each type of culture medium. Images of the wells were captured at the beginning of the experiment and after 24 h on an inverted microscope. All experiments were repeated three times.

### Immunohistochemical staining

In total, 40 formalin-fixed, paraffin-embedded (FFPE) PCa samples were processed for paraffin-embedded sections and used for staining with AZGP1 (Proteintech, 13399-1-AP; 1:200) antibodies, which were dissolved in Immunol Staining Primary Antibody Dilution Buffer (Beyotime, P0103). The detection system utilized was a secondary antibody labeled with the streptavidin–biotin kit (Absin). The histoscore (H-score) was determined as follows: for each tissue sample, the percentages of regions with negative (N%), weakly positive (WP%), moderately positive (MP%), and strongly positive (SP%) staining were determined on the basis of staining intensity; the H-score was then computed by N% × 0 + WP% × 1 + MP% × 2 + SP% × 3.

### Lactic acid, basal OCR, and ECAR detection

First, the lactic acid (LA) content was measured using the LA assay kit. Cells were suspended in physiological saline and subjected to ultrasonic processing for 10 s using the Lawson UP-400 ultrasonic homogenizer to lyse the cells. After centrifugation, the supernatant was transferred to a fresh 1.5-mL tube and mixed with the LA assay reagent. After incubating at 37 °C for 10 min, the termination solution was added and a 530 nm enzyme-linked immunosorbent assay (ELISA) reader was used for detection.

Using the ECAR assay kit, cells were seeded into Nunc 96-well transparent black plates 2 days prior to the experiment. Then, the cells were incubated at 37 °C in a CO_2_-free incubator for 2 h to eliminate CO_2_ interference from the cell culture environment. Subsequently, the pH-sensitive BBcellProbe P61 fluorescent probe provided with the kit was prepared in a diluted buffer and added to the cells to analyze ECAR. The plate was read kinetically for 2 h at 37 °C using the MD SpectraMax i3x microplate reader (every 3 min; ex. 488/em. 585). ECAR was calculated on the basis of the slope of the kinetic curve, as described in the instruction manual.

Basal OCR was measured using an oxygen consumption rate assay kit from Cayman Chemical. The cell treatment was identical to that used in the ECAR assay. After adding the oxygen fluorescent probe and incubation at 37 °C for 30 min, an oxygen blocking solution was added and left at 37 °C for 5 min before testing. The plate was then read kinetically for 2 h at 37 °C using the MD SpectraMax i3x microplate reader (every 3 min; ex. 460/em. 603). The basal OCR levels of the cells were calculated on the basis of the slope of the kinetic curve.

### Bisulfite conversion and methylation-specific PCR

Genomic DNA was isolated from cell lines using the Genomic DNA Mini Preparation Kit with Spin Column (Beyotime Biotechnology, D0063) according to the manufacturer’s instructions. Genomic DNA spiked with 1% unmethylated lambda DNA (Thermo) was converted by bisulfite and purified with the EZ DNA Methylation-Direct Kit (Beyotime Biotechnology, D0033). PCR amplifications were performed using 2× Taq Master Mix (Vazyme Biotech, China).

The design of primers specific for the methylated AZGP gene promoter were as follows:

**Table:** 

MF	AGGTTAATGGGAGTGTTAGGAC (GC: 45.5%; Tm: 54.7 ℃)
MR	CCTCCTACCCACTAACATCCT (GC: 52.4%; Tm: 55.1 ℃)
UF	AGGTTAATGGGAGTGTTAGGAT (GC: 40.9%; Tm: 54.4 ℃)
UR	CCTCCTACCCACTAACATCCT (GC: 52.4%; Tm: 55.1 ℃)

The amplification conditions were set as follows: an initial 1-min denaturation at 94 °C, followed by 35 cycles of 30 s at 94 °C, 45 s at 50 °C, and 1 min at 72 °C, with a final 10-min extension at 72 °C. The PCR products were then assessed using a 1.5% agarose gel and visualized through ethidium bromide staining.

### Lentiviral transduction

Lentivirus was produced by transfecting HEK293T cells for each stable cell line construction. Briefly, about 10 μg target plasmid (PGMLV-CMV-H_AZGP1-3 × Flag-EF1-ZsGreen1-T2A-Puro for human *AZGP1* and pcDNA3.1-Azgp1-EGFP for mouse *Azgp1*), 7.5 μg packaging helper plasmid (psPAX2, Addgene no. 12260), 5 μg envelope helper plasmid (pMD2.G, Addgene no. 12259) and 80 μl of 1 mg/ml polyethylenimine (PEI) were mixed and incubated for 15 min. The mixture was added to HEK293T cells grown in 10 cm plates to 80–90% confluence. After 8 h, the medium was replaced with fresh complete medium. The virus was collected after transfection for 24 h and 48 h. For PC3 or RM-1 cell infection, the mixture of virus, medium, 8 μg/ml polybrene, and 10 mM HEPES were incubated for 24 h. Infected PC3 or RM-1 cells were selected for ZsGreen1+ or EGFP+ cell sorting after 4 days.

### In vivo metastasis model

For the establishment of bone metastasis models, male BALB/c-nu or C57BL/6 mice (6–8 weeks old) were randomly divided into two groups, respectively. The use of laboratory mice was approved by the Institutional Animal Care and Use Committee (IACUC) of Nanjing Medical University (approval no. IACUC-2505054).

PC3-luc cells stably overexpressing *AZGP1* (OE-*AZGP1* group) or empty vector-transfected PC3-luc cells (OE-NC group) were harvested in the logarithmic phase and resuspended in serum-free Dulbecco’s modified Eagle medium (DMEM) at 5 × 10^5^ cells/100 μL. Subsequently, 200 μL of cell suspension was slowly injected into the tail veins of BALB/c-nu mouse. Lung metastasis was monitored on day 14 with the IVIS Lumina Series II In Vivo Imaging System for fluorescence intensities measurement. Images were processed with LivingImage software (v4.4), and data were analyzed through calculating and comparing the average fluorescence intensities among certain areas in the thorax. Mice were sacrificed after in vivo imaging.

RM-1-luc cells stably overexpressing *Azgp1* (OE-*Azgp1* group) or empty vector-transfected RM-1-luc cells (OE-NC group) were harvested in the logarithmic phase and resuspended in serum-free DMEM at 1 × 10^5^ cells/10 μL. Under anesthesia, 10 μL of cell suspension was slowly injected into one of the tibial medullary cavities of the C57BL/6 mouse, with the injection site disinfected and pressed post-operation. Bone metastasis was monitored on day 14 with Bruker In-Vivo FX PRO for fluorescence intensities measurements. Images were processed with Bruker Molecular Imaging software (v7.2.1.22758) and data were analyzed through calculating and comparing the average fluorescence intensities among certain areas at the tibia. Mice were sacrificed after in vivo imaging.

### Statistical analysis

All statistical analyses were performed using R software (v4.0.5). The Wilcoxon rank-sum test for continuous data and Fisher’s exact test for categorical variables were employed to assess differences between groups. Pearson correlation analysis was utilized for correlation analysis. The GSVA package was used to calculate ssGSEA scores. Kaplan–Meier plots were constructed using the Kaplan–Meier *survival* package. All analyses were two-tailed, with statistical significance set at *P* < 0.05.

## Results

### Establishment of the PCa metastasis prediction model

**Fig. 1:**
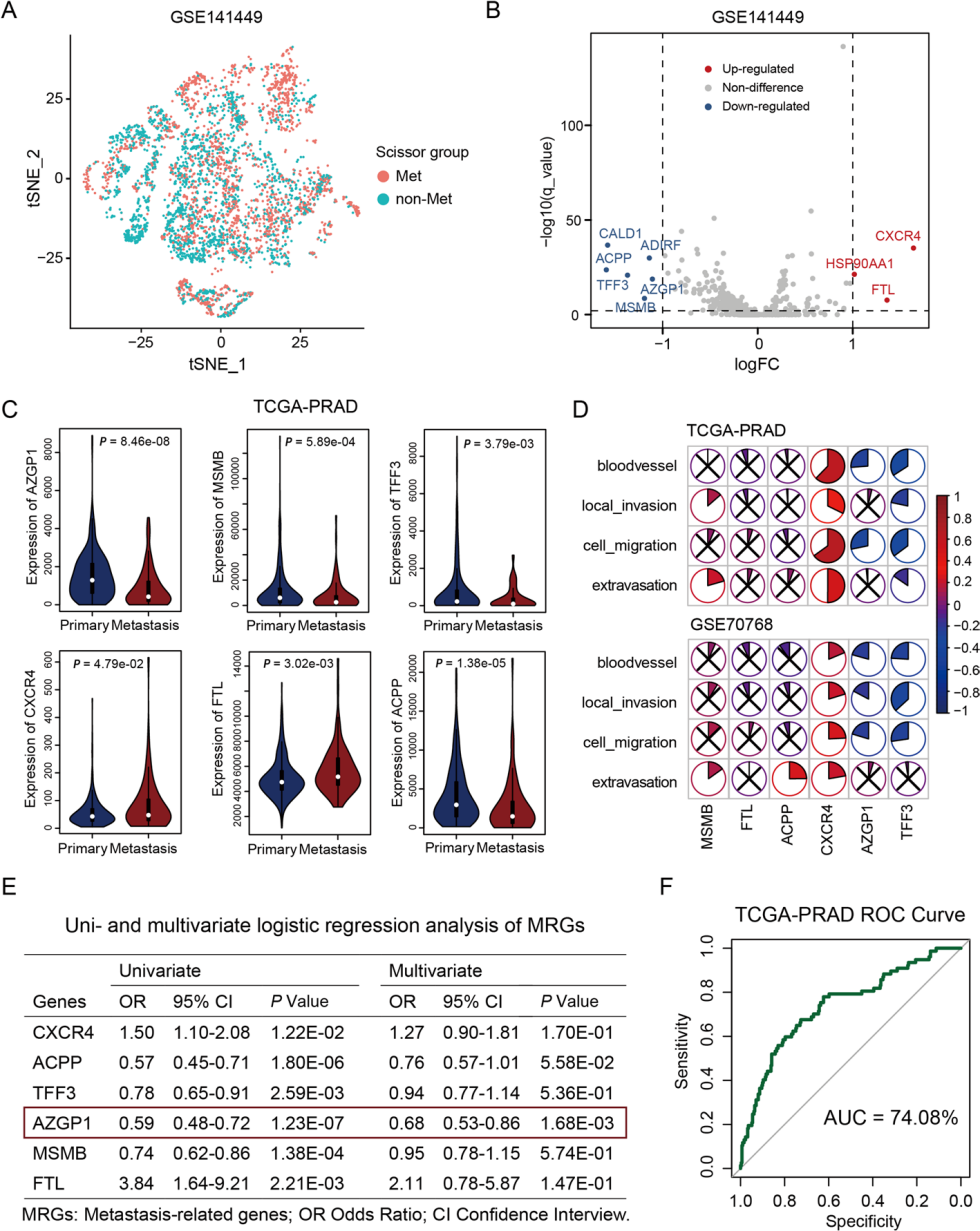
Identification of metastatic subgroups and establishment of models. ** A** The TSNE plots of cells from patients with cells colored based on the LM/Primary cell types. **B** Volcanic plot for differential expression analysis between LM and Primary groups. **C** Violin plots of six significantly differentially expressed genes in TCGA metastatic and non-metastatic patients. **D** The pie plot showed the correlation of gene expression and ssGSEA scores of four pathways related to metastasis, including angiogenesis, local invasion, cell migration, and distant infiltration. **E** Uni-an multivariate logistic regression model of PCa metastasis. **F** The AUC plot of metastasis score.

First, single-cell transcriptome data were preprocessed, including quality control and removal of doublets, resulting in 36,424 cells. Cell types were annotated using the SingleR package and with previously described cell-specific markers. Ultimately, nine cell types were identified: luminal epithelial cells (*AR*), T cells (*CD3E*), B cells (*MS4A1*), mast cells (*KIT*), monocytes (*CD14*), endothelial cells (*CD31*), fibroblasts (*ACTA2*), basal cells (*KRT19*), and other epithelial cells. The process and results of quality control and general cell type annotation have been reported in our previous study which utlized the same dataset [6]. *Scissor* was then used to annotate cells with metastatic advantages, dividing them into two groups: metastatic (Met) and non-metastatic (non-Met) subpopulations (Fig. 1A).

Next, we selected malignant epithelial cells and performed differential expression analysis between cells with and without metastatic advantage to discover metastasis-related markers (Fig. 1B). These markers included *CALD1*, *ADIRF*, *ACPP*, *TFF3*, *AZGP1*, *MSMB*, *CXCR4*, *HSP90AA1*, and *FTL*. Six of these genes: *AZGP1*, *MSMB*, *TFF3*, *CXCR4*, *FTL*, and *ACPP*, named MRGs, have been validated by the TCGA-PRAD cohort (*AZGP1*: *P* = 8.46 × 10^–8^, *MSMB*: *P* = 5.89 × 10^–4^, *TFF3*:* P* = 3.79 × 10^–3^, *CXCR4*:* P* = 4.79 × 10^–2^, *FTL*:* P* = 3.02 × 10^–3^, and *ACPP*:* P* = 1.38 × 10^–5^; Fig. 1C). Then, we correlated the expression of these genes with ssGSEA scores of four pathways related to metastasis, including angiogenesis, local invasion, cell migration, and distant infiltration. The results indicated a significant negative correlation between *AZGP1* expression and tumor metastasis, while *CXCR4* showed a significant positive correlation (Fig. 1D).

On the basis of the discovery of MRGs, we utilized univariate and multivariate logistic regression to establish a PCa metastasis model (Fig. 1E). The receiver operating characteristic (ROC) curve of this model exceeded 70% (Fig. 1F). The model indicates that the *AZGP1* gene can serve as a robust independent predictor of metastasis. In addition, we employed an independent set of single-cell data from Tuong et al. [4] and used the *Scissor* method to identify tumor cells with metastatic potential. By comparing *AZGP1* expression between Met and non-Met subgroups, we further confirmed our findings that tumor cells with lower *AZGP1* expression exhibited metastatic capability (*P* = 2.289 × 10^–7^; Supplementary Fig. 1).

### *AZGP1* expression is associated with the progression and prognosis of PCa

**Fig. 2:**
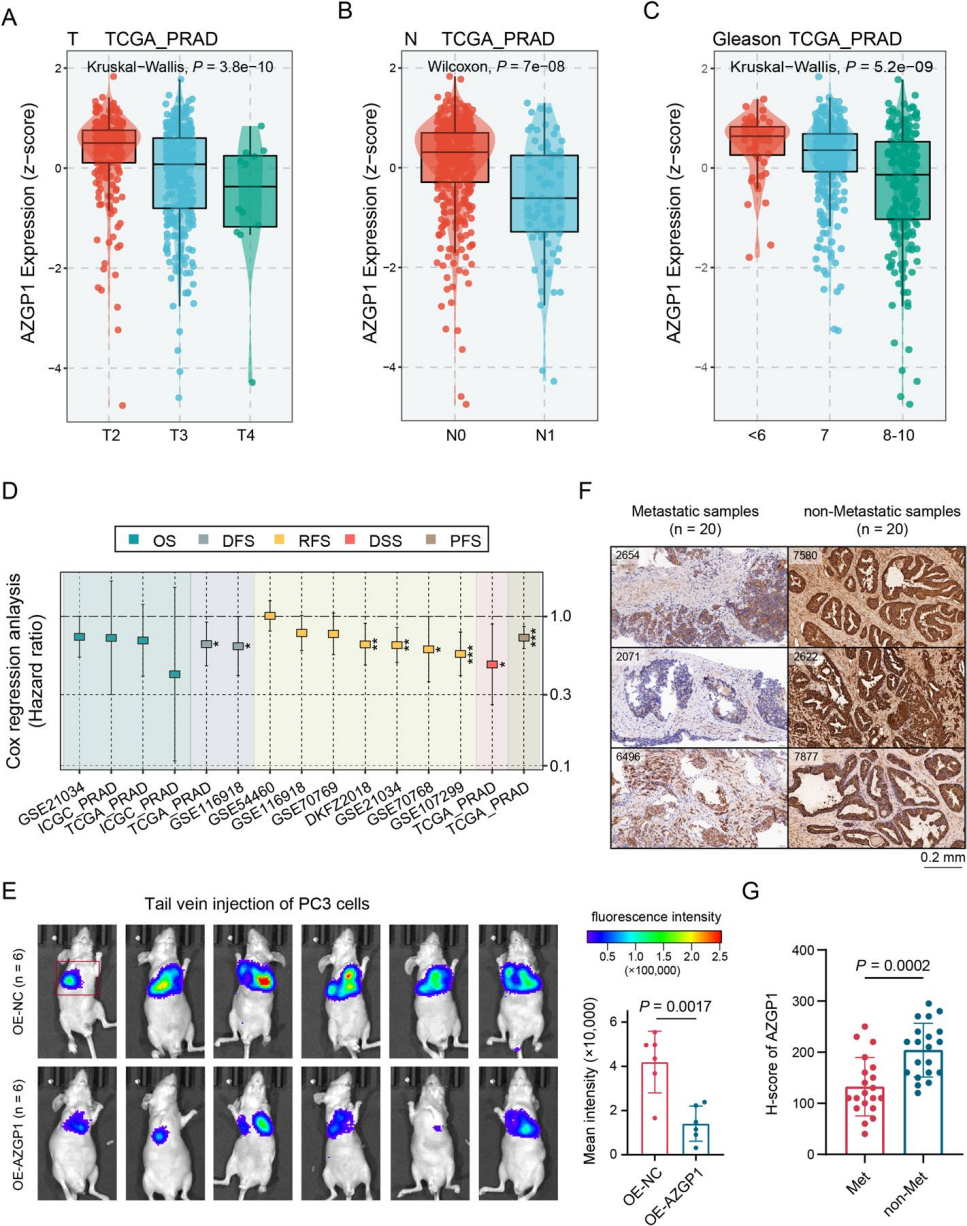
Lower *AZGP1* expression facilitates the progression and prognosis of PCa. **A** Boxplot of the differences in *AZGP1* expression among PCa samples with T2, T3, and T4 stages in the TCGA-PRAD dataset.** B** Boxplot of the differences in *AZGP1* expression between N0 and N1 PCa samples in the TCGA-PRAD dataset. **C** Boxplot of the differences in *AZGP1* expression among PCa samples with different Gleason scores in the TCGA-PRAD dataset. **D** Forest plot visualizing the meta-analysis of nine bulk datasets based on the Cox regression analysis of *AZGP1* expression. **E** In vivo imaging of six OE-*Azgp1* and six NC mice (left) with tail vein injections of PC3 cells. Comparison of the average immunofluorescence intensity of the metastatic regions (right). **F** Representative images of immunohistochemical staining for AZGP in metastatic and non-metastatic PCa tissue samples. **G** Scatter plots comparing the histoscores (H-score) of the immunohistochemical staining between 20 metastatic and 20 non-matastatic PCa tissue samples. *P < 0.05, **P < 0.01, ***P < 0.001, ****P < 0.0001

Utilizing multiple datasets, including GSE21034, DKFZ2018, and TCGA-PRAD cohorts, we compared the expression levels of *AZGP1* across different T stages, N status, Gleason scores, and tumor mutation burdens (TMB). The results demonstrated that lower mRNA expression of *AZGP1* was associated with advanced T stage, N stage, and Gleason score (*P*_(all)_ < 0.05; Fig. 2A–C; Supplementary Fig. 2A, B). In addition, patients with low *AZGP1* expression exhibited a significantly higher TMB compared with those with high expression (*P* = 8.9 × 10^–7^; Supplementary Fig. 2C).

Analysis based on multiple datasets revealed a significant association between *AZGP1* expression and patient outcomes, indicated by single-gene Cox analysis for disease-free survival (DFS), recurrence-free survival (RFS), and progression-free survival (PFS) (Fig. 2D). Lower *AZGP1* expression was correlated with poorer PFS (*P* = 0.0023; Supplementary Fig. 2D). After establishing the lung and bone metastasis models, we compared the fluorescence intensities of the metastases between overexpressing *AZGP1* and the NC group. The results showed that the metastatic capability of the OE-*AZGP1* group was significantly lower than that of the NC group (*P* = 0.0017 and 0.0217 for lung and bone metastases, respectively; Fig. 2E; Supplementary Fig. 2E). Immunohistochemical staining with AZGP1 showed significantly higher H-scores in non-metastatic than in metastatic tissues (*P* = 0.0002; Fig. 2F, G). In summary, *AZGP1* serves as a potential biomarker for PCa metastasis, with decreased expression suggesting an increased likelihood of metastasis and recurrence, leading to a decreased overall survival.

### Reduced expression of *AZGP1* leads to tumor progression and metastasis

**Fig. 3:**
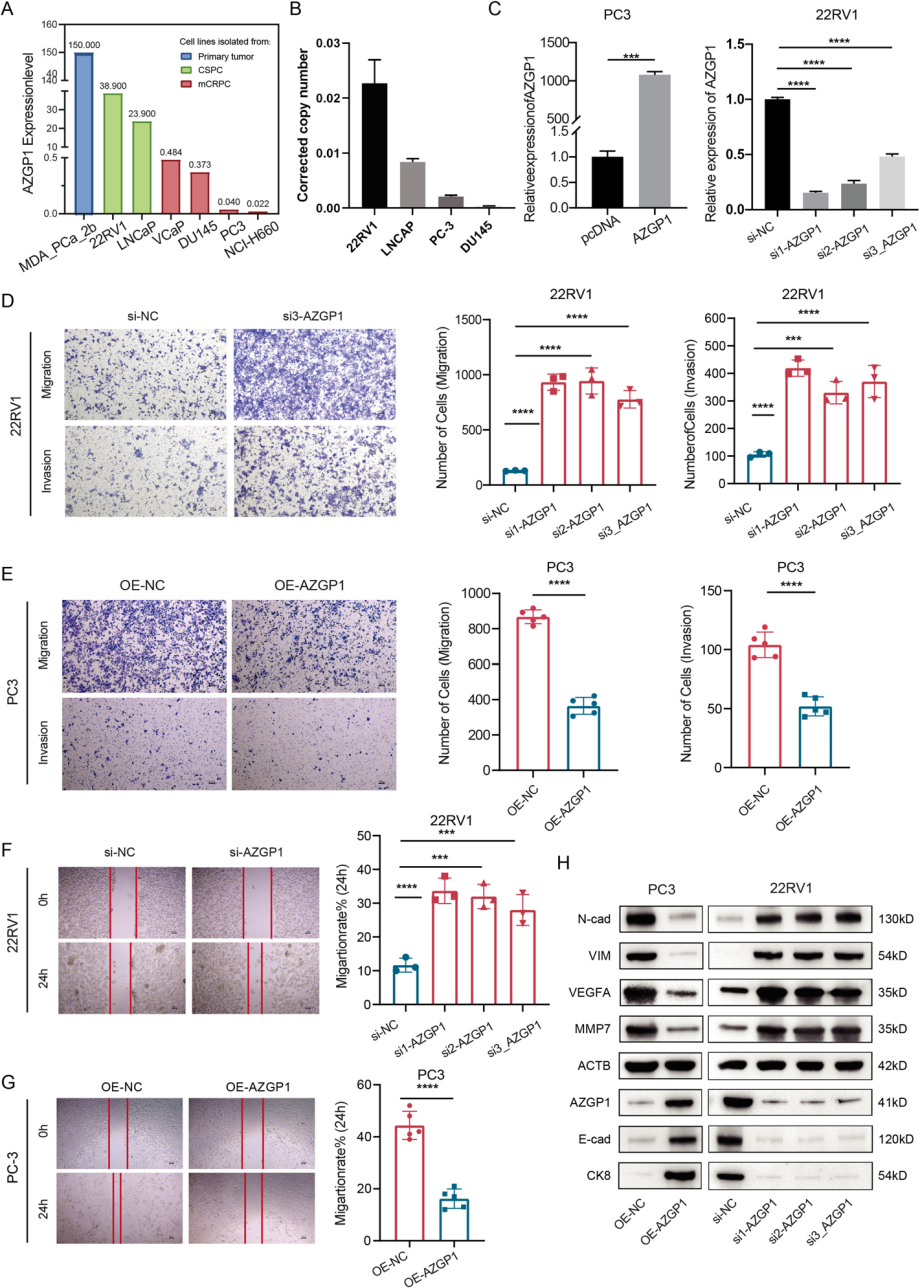
*AZGP1* regulates the cell proliferation and migration of PCa cells.** A** Relative expression of *AZGP1* in seven prostate epithelial cell lines in the Cancer Cell Line Encyclopedia (CCLE) database. **B** Relative expression of *AZGP1* in five human prostate epithelial cell lines: 22RV1, LNCaP, PC3, and DU145. **C** qRT-PCR analyses of *AZGP1* mRNA in PC3 cells treated with negative control (NC) or *AZGP1* pcDNA. qRT-PCR analyses of *AZGP1* mRNA in 22RV1 cells treated with negative control (NC) or *AZGP1* siRNA (siRNA1, siRNA2, and siRNA3). **D** Transwell assays assessed cell migration and invasion in 22RV1 cells upon *AZGP1* knockdown.** E** Transwell assays assessed cell migration and invasion in PC3 cells upon *AZGP1* overexpression. **F**, **G** Wound healing assays evaluated the migration of 22RV1 and PC3 cells. **H** Western blot analysis of the protein levels of N-cadherin, E-cadherin, VEGFA, MMP7, and AZGP1 after *AZGP1* was overexpressed in PC3 cells and was knocked down in 22RV1 cells. *P < 0.05, **P < 0.01, ***P < 0.001, ****P < 0.0001

Using the Xena to analyze cancer cell line encyclopedia (CCLE) database, we collected three types of PCa cell lines, including primary tumor cells (including MDA PCa ab), castration-sensitive PCa (CSPC; including 22RV1 and LNCaP), and metastatic castration-resistant PCa (mCRPC; including VCaP, DU145, PC3, and NCI-H660). Comparing the expression of *AZGP1* in each cell line, we observed that, consistent with tissue sample results, *AZGP1* expression was highest in primary tumors, lower in castration-sensitive PCa, and lowest in metastatic castration-resistant PCa (Fig. 3A). To validate these findings, we performed RT-qPCR for *AZGP1* in four cell lines, confirming that the expression in metastatic cell lines (PC3 and DU145) was low, while primary tumor cell lines (LNCaP and 22RV1) showed higher expression (Fig. 3B). Next, we constructed PCa cell lines with overexpression or knockdown of *AZGP1*, and RT-qPCR was performed to verify the efficiency of overexpression and knockdown (Fig. 3C). Knockdown of *AZGP1* was carried out in the 22RV1 line, and the migration and invasion capacity of tumor cells in Transwell assays were significantly enhanced after knockdown of *AZGP1* (Fig. 3D). In contrast, overexpression of *AZGP1* reduced the migration and invasion of PC3 cells (Fig. 3E). The results were consistent with wound healing assays that linked *AZGP1* expression with reduced migration capacity (*P*_all_ < 0.05; Fig. 3F, G). Subsequently, western blot analysis was conducted to determine the changes in the levels of tumor metastasis-related proteins, including E-cadherin, N-cadherin, VEGFA, and MMP7, which showed a significant expression increase of these proteins upon *AZGP1* knockdown and a decrease upon *AZGP1* overexpression (*P*_all_ < 0.05; Fig. 3H).

### Decreased expression of *AZGP1* activates the glycolytic pathway

**Fig. 4:**
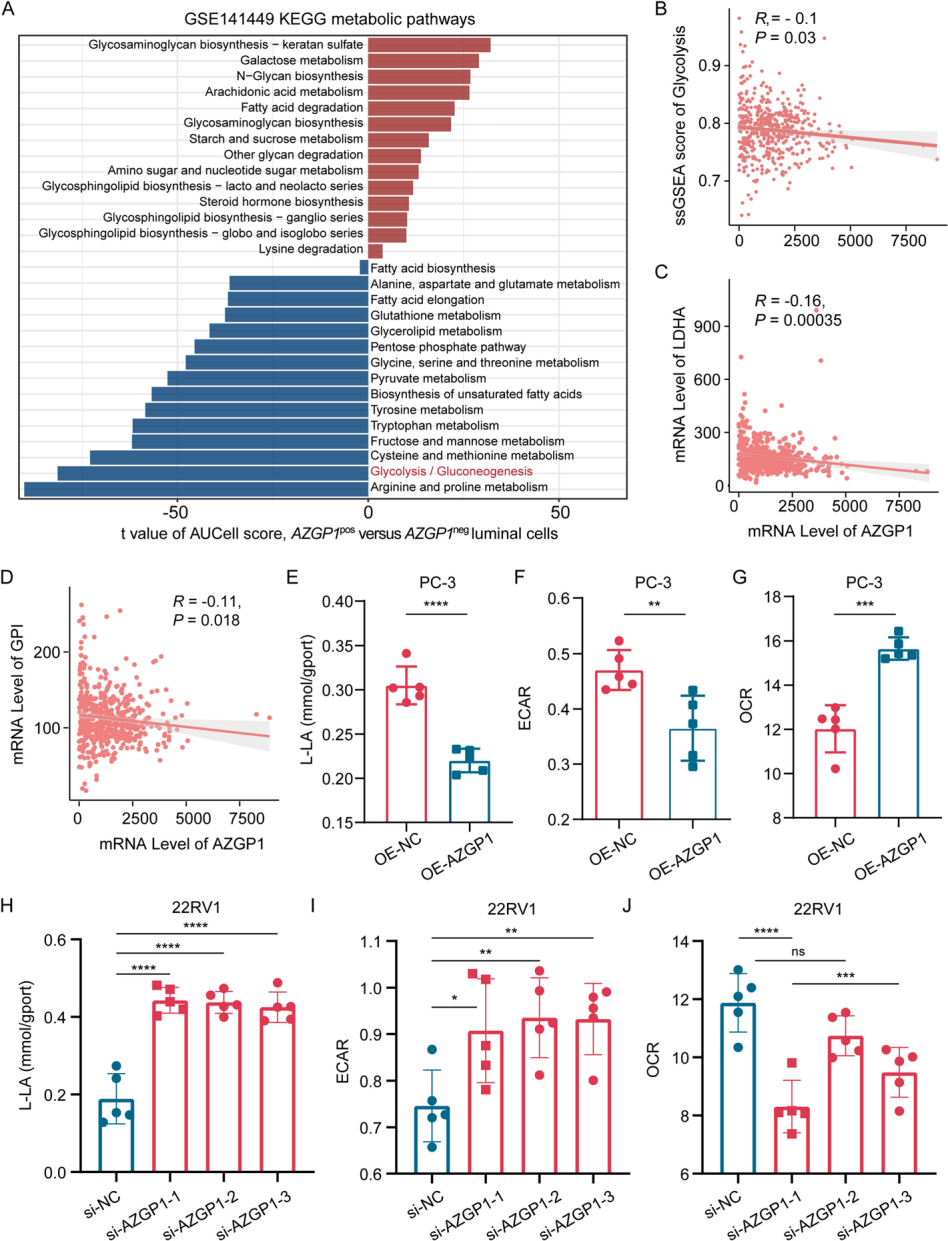
Decreased expression of *AZGP1* activates the glycolytic pathway. **A** Bar plots showing the differential enrichment of KEGG metabolism pathways between LM and primary cancer cells derived from *scMetabolism* analyses in scRNA-seq data. **B** Correlation plot of *AZGP1* expression with the ssGSEA score of the glycolysis pathway. **C**, **D** Correlation plots of *AZGP1* expression with expression of the glycolytic enzymes *LDHA* and *GP1*. **E–G** Boxplot of lactic acid production, basal OCR, and ECAR detection in PC3 and OE-*AZGP1* PC3 cells. **H**–**J** Boxplot of lactic acid production, basal OCR, and ECAR detection in 22RV1 and si-22RV1 cells. *P < 0.05, **P < 0.01, ***P < 0.001, ****P < 0.0001

Metabolic analysis of single-cell data reveals that tumor cells with lower *AZGP1* expression exhibit significantly higher glycolytic capacity (Fig. 4A). The ssGSEA score of the glycolysis pathway significantly decreased with the increase of *AZGP1* expression (*R* = −0.1, *P* = 0.03; Fig. 4B). Correlation analysis revealed a significant negative correlation between *AZGP1* expression and glycolytic enzymes, including *LDHA*, *GP1, PFKFB3*, *SLC2A1*, *PDK1*, and *PGK1* (*R* = −0.16, *P* = 0.00035; *R* = −0.11, *P* = 0.018; *R* = −0.11, *P* = 0.011; *R* = −0.09, *P* = 0.041; *R* = −0.23, *P* = 3.4 × 10^–7^; and *R* = –0.13, *P* = 0.0048, respectively; Fig. 4C, D; Supplementary Fig. 3A–D). Then, lactate production was measured in control and OE-*AZGP1* PC3 cells, and the result showed that after overexpression of *AZGP1*, l-lactic acid (L-LA) production in the PC3 cell line significantly decreased, indicating a significant inhibition of glycolytic capacity (Fig. 4E). Then, we measured the ECAR in PC3 and OE-*AZGP1* PC3 cells. The results showed that glycolytic capacity was significantly decreased in OE-*AZGP1* PC3 cells but increased in the PC3 group (Fig. 4F). OCR assays, which reflect mitochondrial respiration, displayed an opposite tendency to those of ECAR (Fig. 4G). *AZGP1* knockdown was performed on the 22RV1 cell line, and the levels of L-LA production, ECAR, and OCR before and after knockdown were detected. The results showed that after the knockdown of *AZGP1* expression, the production of L-LA significantly increased (*P* < 0.0001; Fig. 4H), glycolytic capacity significantly increased (*P* < 0.001; Fig. 4I), and mitochondrial respiration capacity significantly decreased (*P* < 0.0001; Fig. 4J). The results indicated that loss of *AZGP1* expression led to elevation of the glycolytic pathway, promoting tumor proliferation and metastasis.

### Differential promoter region methylation regulates *AZGP1* expression in PCa metastasis

**Fig. 5:**
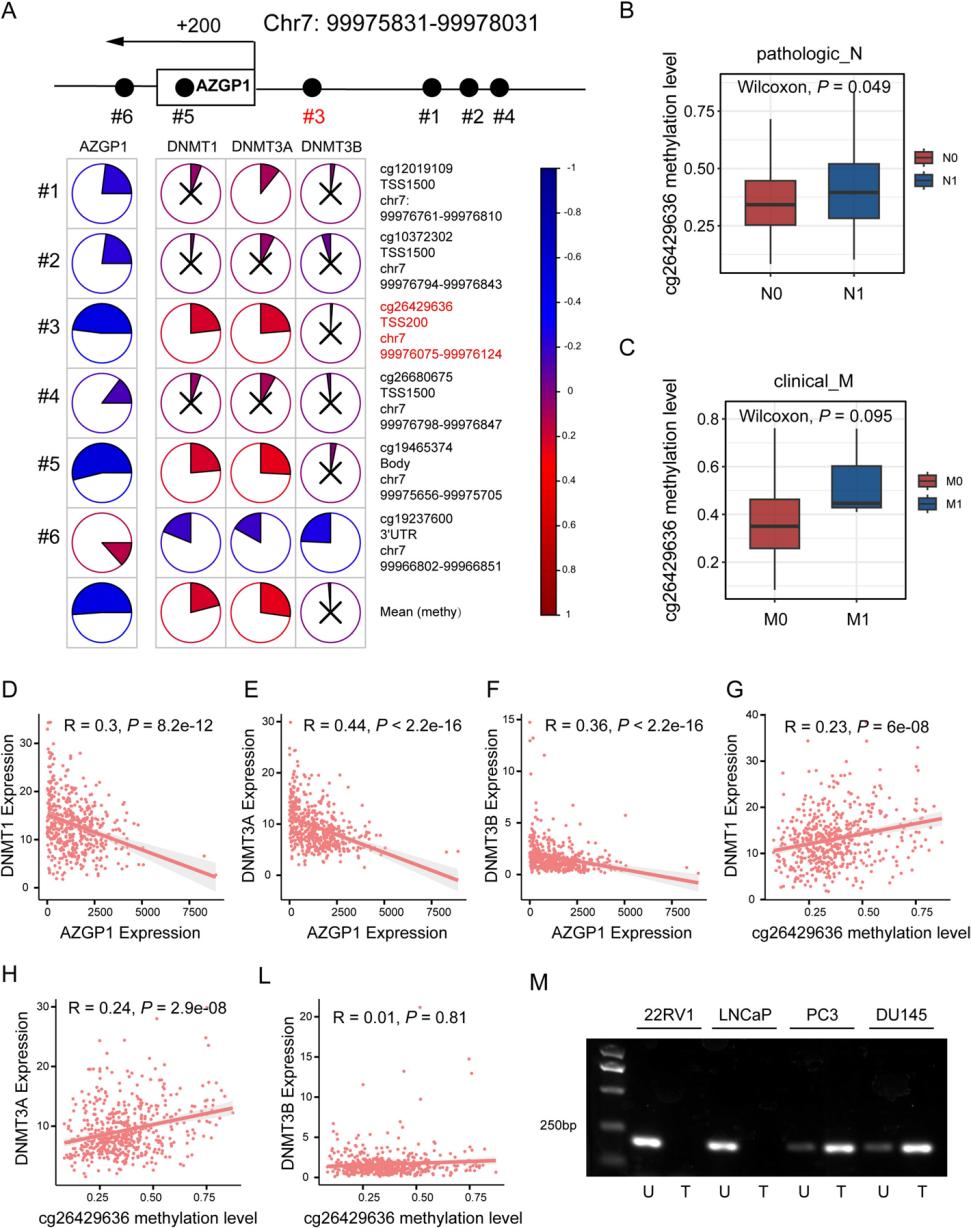
Differential promoter region methylation regulates *AZGP1* expression in PCa metastasis.** A** The regulatory model diagram of *AZGP1* promoter methylation and gene expression (upper). Correlation of DNA methylation changes at promoter and gene expression levels (left lower). Correlation of DNA methylation changes at promoter and methyltransferases (right lower). **B** Boxplot of cg26429636 methylation level between N0 and N1 PCa in the TCGA-PRAD database. **C** Boxplot of cg26429636 methylation levels between M0 and M1 PCa in the TCGA-PRAD database. **D–F** Scatter plot of correlations between the mRNA level of *AZGP1* and methyltransferases, including DNMT1, DNMT3A, and DNMT3B. **G–L** Scatter plot of correlations between cg26429636 methylation level and methyltransferase expression, including DNMT1, DNMT3A, and DNMT3B. **M** Methylation-specific PCR (MSP) analysis of the methylation status of *AZGP1* shows aberrant methylation in 22RV1, LNCaP, PC3, and DU145 cell lines. “M” and “U” represent MSP results using primer sets for methylated and unmethylated *AZGP1* genes, respectively. *P < 0.05, **P < 0.01, ***P < 0.001, ****P < 0.0001

Mutation rates were significantly different between *AZGP1*-high and *AZGP1*-low samples (*P*_all_ < 0.05; Supplementary Fig. 3E, F). Further comparison of the mutation spectra revealed several high-frequency mutations, including *TP53*, *STAT4*, *VPS13D*, and *TAF1L* (*P*_all_ < 0.05; Supplementary Fig. 3G). There was no significant difference in copy number variation between the two groups of patients (Supplementary Fig. 3H). We performed a correlation analysis between gene promoter methylation levels and mRNA expression. The results indicated that cg26429636, located in the 200 bp upstream region of the promoter, showed a significant negative correlation with *AZGP1* expression (Fig. 5A). Moreover, the methylation levels at the altered sites were significantly higher in patients with lymph node metastasis compared with non-metastatic patients (*P* = 0.049; Fig. 5B). Although no significant difference was observed between patients with distant metastasis and non-metastatic patients, a trend was noted, potentially attributed to the limited number of metastatic samples (*P* = 0.095; Fig. 5C). Furthermore, there was a significant negative correlation between the expression level of AZGP1 and methyltransferase genes: *DNMT1*, *DNMT3A*, and *DNMT3B* (*R* = 0.3, *P* = 8.2 × 10^–12^; *R* = 0.44, *P* < 2.2 × 10^–16^; *R* = 0.36, *P* < 2.2 × 10^–16^, respectively; Fig. 5D–F). Positive correlations between the methylation levels at cg26429636 and the expression level of methyltransferase genes *DNMT1* and *DNMT3A* were also identified (*R* = 0.23, *P* = 6.0 × 10^–8^; *R* = 0.24, *P* = 2.9 × 10^–8^, respectively; Fig. 5G-L). We used methylation-specific PCR (MSP) methods to detect the promoter methylation levels of four cell lines (PC3, LNCap, DU145, and 22RV1), and the results showed that in CSPC cell lines, including 22RV1 and LNCaP, promoters were unmethylated, while in mCRPC cell lines, including PC3 and DU145, promoters were partially methylated (*P*_all_ < 0.05; Fig. 5M). In conclusion, methylation is present at the promoter site cg26429636 of the *AZGP1* gene, which we believe is the reason for the decreased gene expression. Promoter methylation may serve as a surrogate marker of *AZGP1* expression and holds diagnostic and prognostic potential.

## Discussion

With the advancement of single-cell sequencing, researchers have made new progress in understanding the metastatic patterns of tumors. During malignant progression, only a subset of tumor cells with metastatic advantages will systematically disseminate within the organism and initiate metastasis. Furthermore, cells possessing these metastatic traits continually proliferate, exacerbating distant tumor metastasis and colonization. Hence, identifying these subsets of cells with metastatic advantages holds significant importance for inhibiting tumor metastasis.

In this study, we hypothesized that within the primary tumor, a population of tumor cells with metastatic potential promotes tumor proliferation, migration, and distant colonization. By integrating single-cell transcriptomics with bulk data from large cohorts, we employed the *Scissor* tool to identify and characterize metastatic-capable tumor cells. Differential analysis between these subpopulations led to the identification of genes associated with metastatic features and the development of a metastatic model, which achieved an AUC of 74.08%. The model scores demonstrated a significant positive correlation with metastasis-associated pathways. Notably, the metastatic model revealed that low expression of *AZGP1* promotes tumor metastasis. Previous studies have indicated that *AZGP1* possesses zinc-binding properties and its expression is reduced in more aggressive cancer lesions, such as PCa, breast cancer, colon cancer, liver cancer, gastric cancer, and lung cancer [7–11]. Numerous single-center studies have indicated that *AZGP1* can serve as a biomarker for PCa and predict biochemical recurrence of the disease [12, 13]. A prospective multicenter phase III validation study conducted by Zhang et al. demonstrated that immunohistochemical expression of *AZGP1* can predict PCa metastasis and mortality [14]. Furthermore, this research team found that low or absent expression of *AZGP1* independently predicts biochemical recurrence and metastasis. Compared with existing prognostic risk models, such as the stratification system designed by Gnanapragasam et al. [15] and the CAPRA-S score [16], AZGP1 offers enhanced discriminative value for metastatic recurrence. Our analysis of multiple, large clinical cohort datasets also showed that *AZGP1* expression correlates with PCa staging and prognosis. The results indicate that patients with low *AZGP1* expression have higher tumor stages, greater tumor malignancy, and are more likely to have metastases, with significantly worse outcomes compared with patients with high *AZGP1* expression. Our study included data from multiple ethnic backgrounds, and previous research had also encompassed results from diverse ethnic backgrounds, demonstrating that the *AZGP1* gene serves as a biomarker for PCa metastasis with ethnic conservancy. Subsequently, we investigated several PCa cell lines and selected four representative ones for our study. The PC3 cell line, derived from human PCa bone metastasis, is poorly differentiated and exhibits moderate metastatic potential [17, 18]. The DU145 cell line, obtained from brain metastasis in a 63-year-old male patient with PCa, is also poorly differentiated, androgen-independent, and possesses strong metastatic potential. The LNCaP cell line, derived from PCa lymph node metastasis, is characterized by high E-cadherin expression and absence of vimentin, reflecting significant epithelial cell properties [19]. The 22RV1 cell line, established from xenografts of the hormone-refractory PCa cell line CWR22 in athymic nude mice, represents another relevant model [20]. Quantitative analysis of *AZGP1* expression across these cell lines revealed the lowest expression in DU145 and the highest in 22RV1, consistent with results from the CCLE database. Invasive and migratory assays, along with assessments of epithelial–mesenchymal transition (EMT)-related proteins in the representative PC3 and 22RV1 cell lines, confirmed our hypothesis: low *AZGP1* expression enhances cell invasion and migration, promoting tumor cell metastasis. Our findings align closely with previous research, validating the strong association between metastatic potential and tumor cell characteristics. AZGP1 thus serves as a biomarker for predicting metastasis, with low *AZGP1* expression indicating high metastatic potential in tumor cells.

To investigate how low *AZGP1* expression mediates tumor metastasis, we performed an in depth analysis of single-cell data and identified significant differences in the metabolic profiles of metastatic versus non-metastatic tumor cells, particularly in glycolysis. We also observed a notable negative correlation between *AZGP1* expression and six glycolytic enzymes. These findings suggest that low *AZGP1* expression may promote glycolytic metabolism, leading to tumor metastasis. Tumor cells utilize glycolysis for metabolic reprogramming, providing sufficient energy and substrates for tumor growth. Moreover, glycolysis-generated lactate and other metabolic byproducts can enhance tumor invasion and metastasis by altering the cellular microenvironment [21, 22]. Glycolytic products activate specific signaling pathways, such as hypoxia-inducible factor-1α (HIF-1α), which further facilitates cancer cell metastatic potential [23]. Studies have shown that glycolysis inhibitors can significantly reduce the metastatic potential of PCa cells. For example, inhibitors of glycolytic enzymes, such as hexokinase, have demonstrated promising antitumor effects in experimental settings [24]. By measuring l-lactic acid production, extracellular acidification rates, and oxygen consumption rates (which reflects mitochondrial respiration) in PC3 cell lines before and after *AZGP1* overexpression, we demonstrated that restoring *AZGP1* expression reduces the glycolytic capacity of tumor cells. These results indicate that low *AZGP1* expression promotes tumor metastasis by modulating glycolysis in cancer cells.

Finally, through integrative multi-omics analysis, we discovered that the transcriptional level of *AZGP1* was influenced by promoter methylation, which significantly reduced mRNA expression. Moreover, by assessing the promoter methylation levels in various cell lines, we demonstrated that measuring promoter methylation levels could serve as a proxy for transcriptional levels. Therefore, we proposed that promoter methylation levels could be used to differentiate patients with metastatic risk. The impact of *AZGP1* promoter methylation on tumors has been reported in other cancers. For example, Hou et al. found that *AZGP1* promoter methylation in uveal melanoma significantly reduced mRNA levels and served as a predictor of melanoma clinical outcomes [25].

In this study, we explored the characteristics of tumor cells with metastatic potential by integrating single-cell and bulk data. We identified that tumors characterized by low *AZGP1* expression exhibited enhanced glycolytic activity, making these cells more prone to metastasis. Our research provides a novel cellular-level interpretation of tumor metastasis. However, current validation of the underlying mechanisms has only been conducted at the cell line level, and validation with large clinical cohorts remains pending. The inference regarding the role of glycolysis in AZGP1-mediated PCa metastasis is partly based on a review and synthesis of existing literature. Further experimental studies are required to establish a direct causal relationship and elucidate the underlying molecular mechanisms. In addition, this study did not establish direct causal relationships between DNMTs, cg26429636 methylation, and PCa metastasis. Further experimental validation is required to confirm these mechanistic links.

In summary, promoter methylation of *AZGP1* leads to reduced transcriptional expression, thereby promoting glycolysis in tumor cells and facilitating metastasis. The detection of *AZGP1* methylation levels offers a valuable reference for dynamic surveillance of PCa metastasis.

## Supplementary Information

Supplementary Material 1

Supplementary Material 2

Supplementary Material 3

Supplementary Material 4

Supplementary Material 5

## Data Availability

All sequencing data used in this study are available publicly: GSE141445 are available at the GEO database. TCGA-PRAD transcriptome data were obtained from Genomic Data Commons data Portal (GDC, https://portal.gdc.cancer.gov). Survival analyses and clinical relevance analyses based on GSE21034, DKFZ2018, and prad_su2c_2015 datasets were conducted using Biomarker Exploration of Solid Tumors database (BEST, https://rookieutopia.hiplot.com.cn/app_direct/BEST/).

## Abbreviations

PCa: Prostate cancer
ECAR: Extracellular acidification rate
OCR: Oxygen consumption rate
AZGP1: Alpha-2-glycoprotein 1
MSP: Methylation-specific PCR
TME: Tumor microenvironment
GDC: Genomic Data Commons
TPM: Transcripts per million
LM: Lymph node metastasis
ssGSEA: Single sample gene set enrichment analysis
MRGs: Metastasis-related genes
VIF: Variance inflation factor
si-NC: Small interfering RNA and negative control RNA
ELISA: Enzyme-linked immunosorbent assay
WB: Western blotting
TMB: Tumor mutation burden
DFS: Disease-free survival
RFS: Recurrence-free survival
PFS: Progression-free survival
L-LA: l-lactic acid
EMT: Epithelial–mesenchymal transition
HIF-1α: Hypoxia-inducible factor-1α
FFPE: Formalin-fixed, paraffin-embedded
scRNA-seq: Single-cell RNA sequencing
