# Supplementary figures and images for: Methylation-induced silencing of AZGP1 enhances prostate cancer metastasis by stimulating tumoral glycolysis
Source: Cell Mol Biol Lett. 2026 Jan 14;31:5. doi: 10.1186/s11658-025-00818-3 (PMC12801908; doi:10.1186/s11658-025-00818-3)

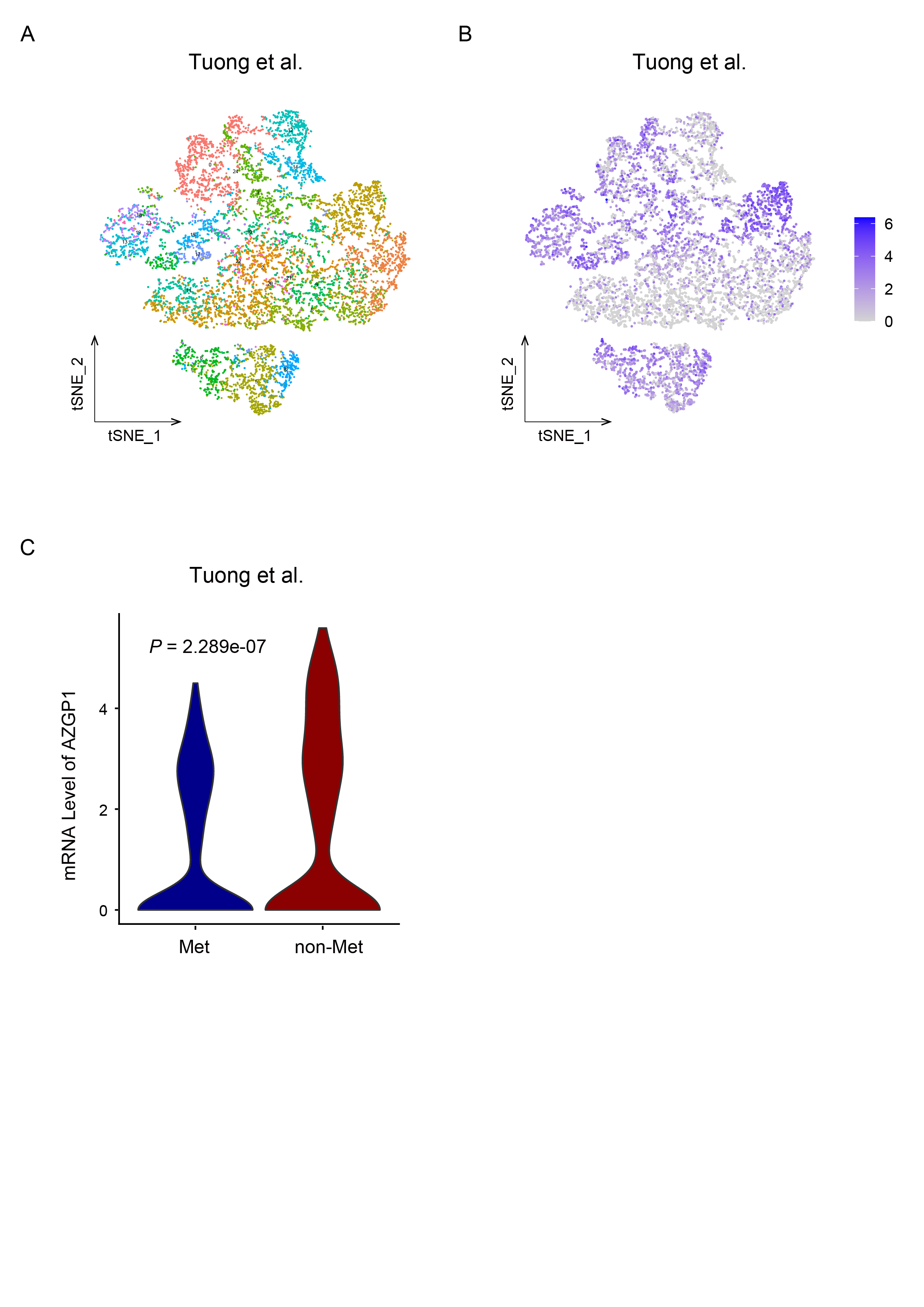

Supplement: Supplementary file 1 — Supplementary Material 1 [file 11658_2025_818_MOESM1_ESM.tif]

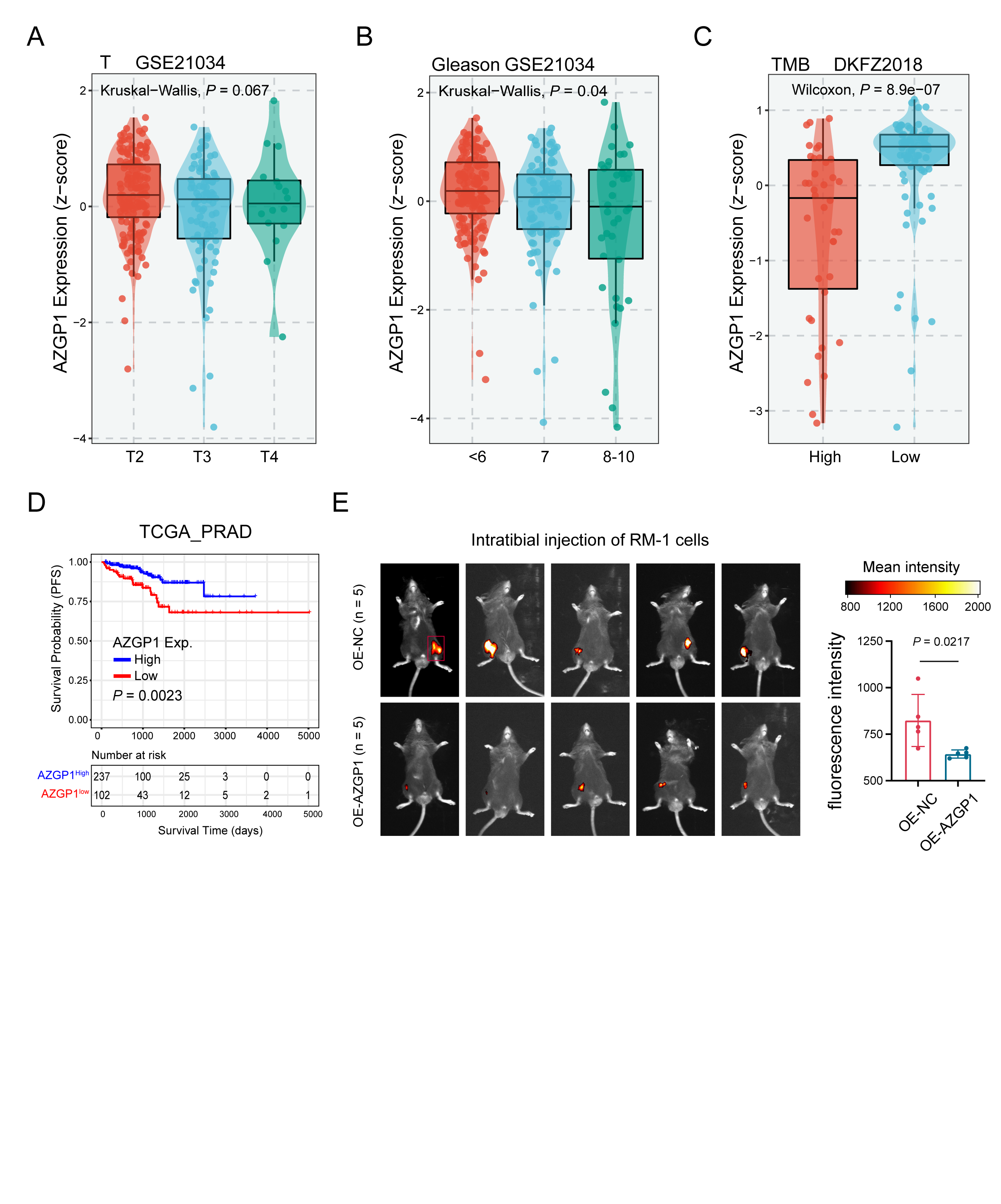

Supplement: Supplementary file 2 — Supplementary Material 2 [file 11658_2025_818_MOESM2_ESM.tif]

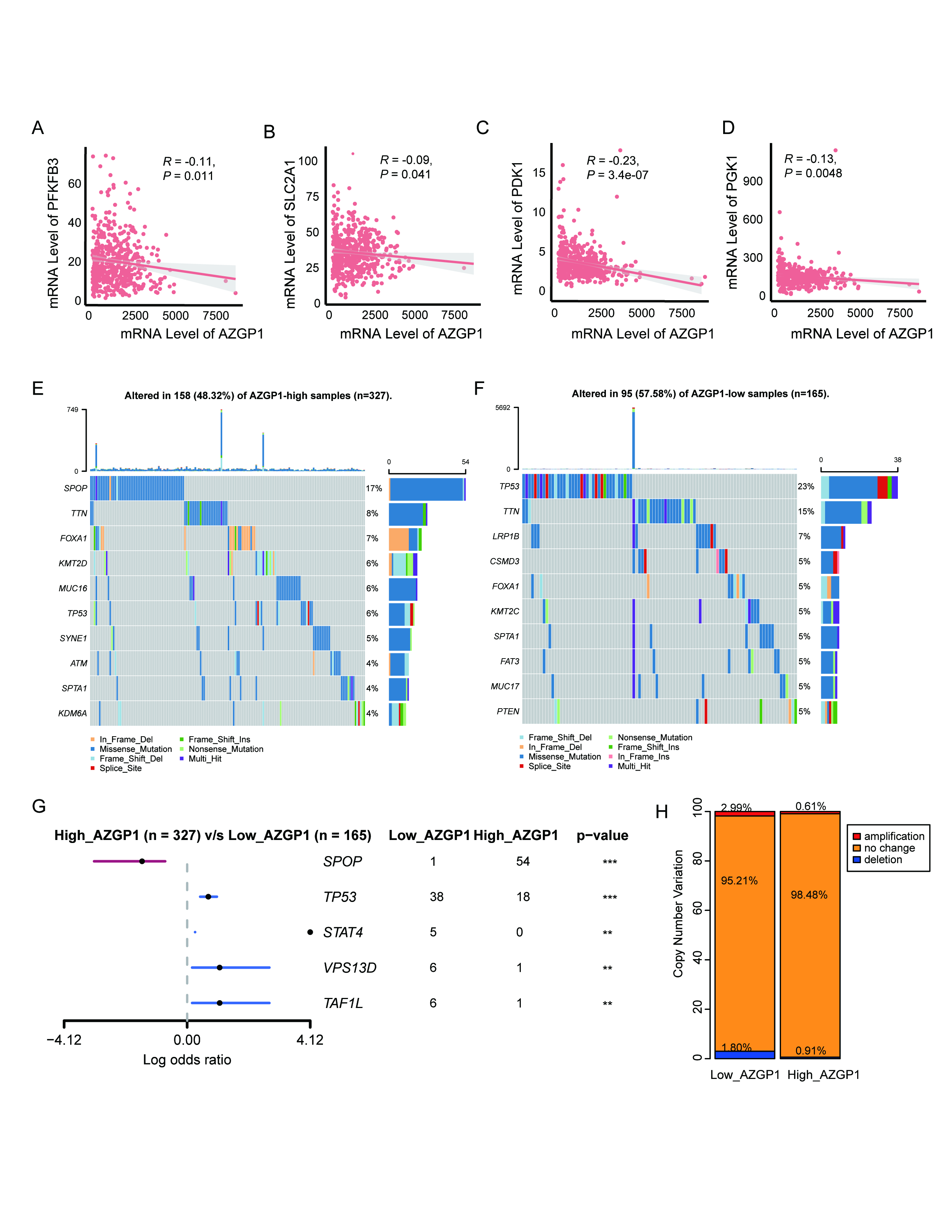

Supplement: Supplementary file 3 — Supplementary Material 3 [file 11658_2025_818_MOESM3_ESM.tif]
