# Supplementary material for: Methylation-induced silencing of AZGP1 enhances prostate cancer metastasis by stimulating tumoral glycolysis
Source: Cell Mol Biol Lett. 2026 Jan 14;31:5. doi: 10.1186/s11658-025-00818-3 (PMC12801908; doi:10.1186/s11658-025-00818-3)

Supplementary Fig4

Raw western blots-OE rep1

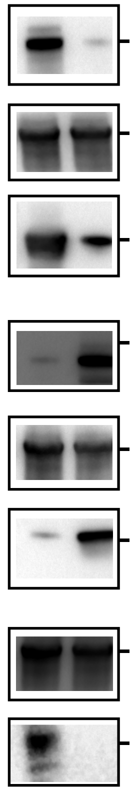

N-CAD, VEGFRA

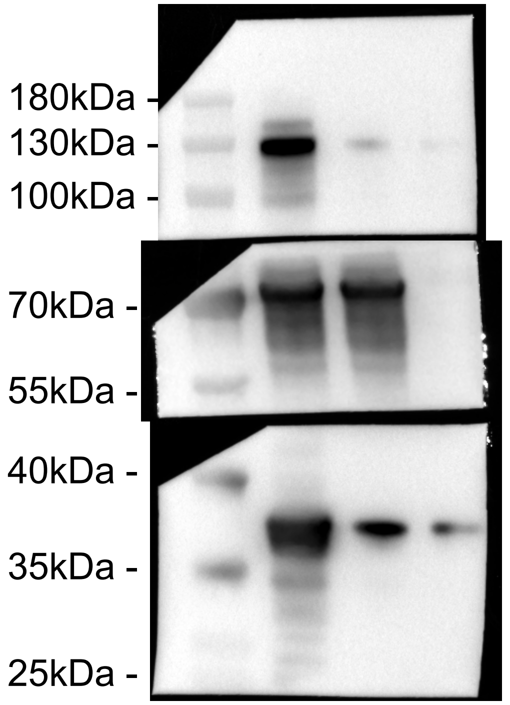

E-CAD, AZGP1

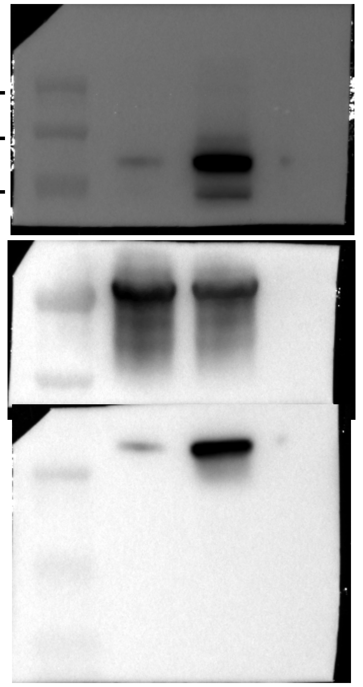

MMP7

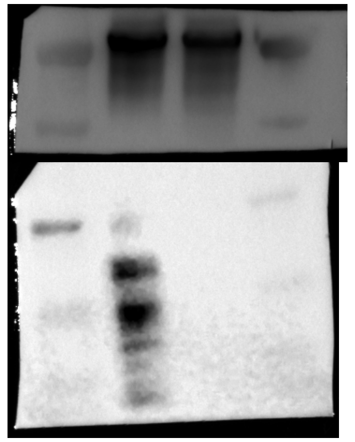

Combination

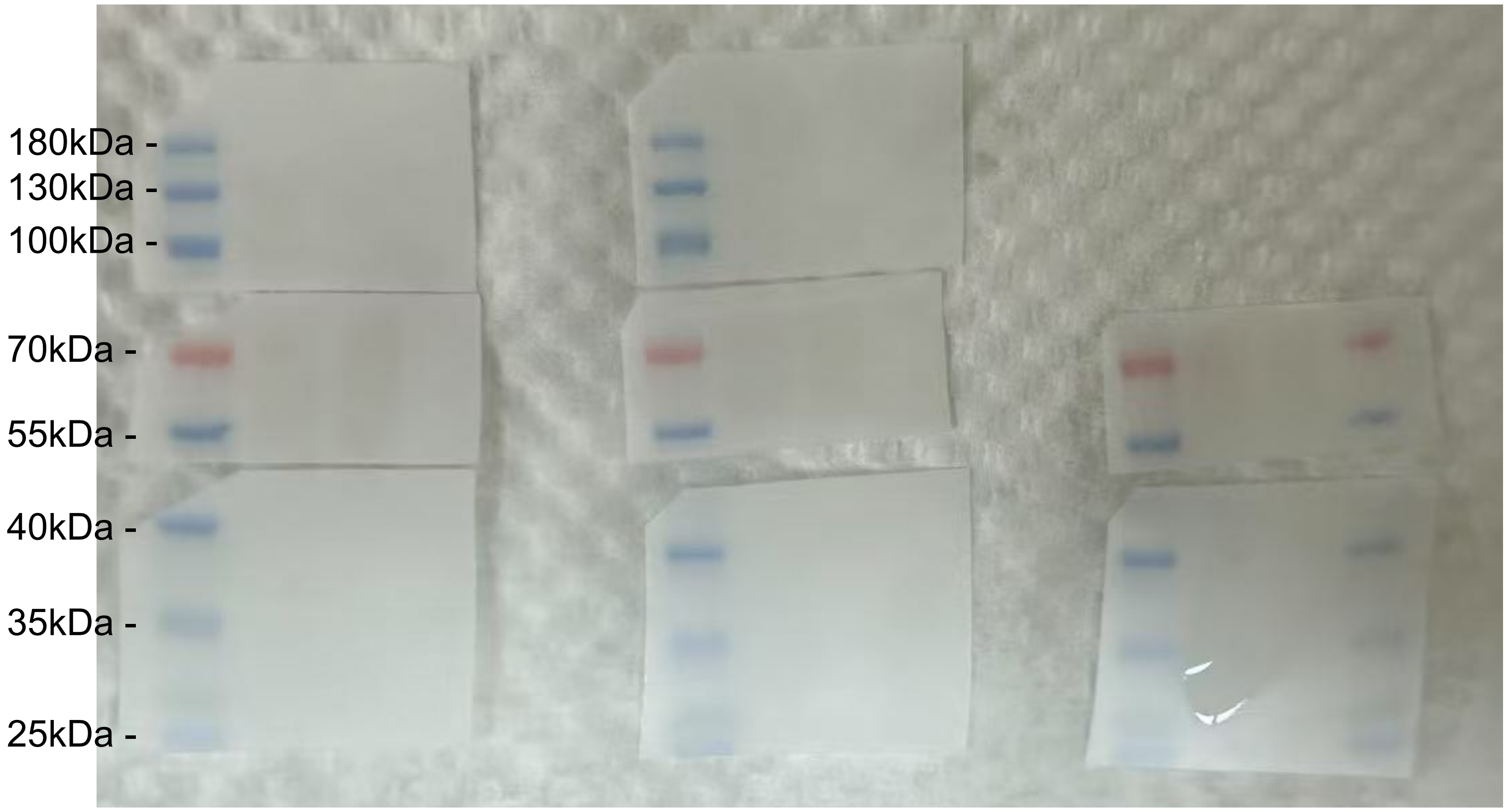

Supplementary Fig4

Raw western blots-OE rep2

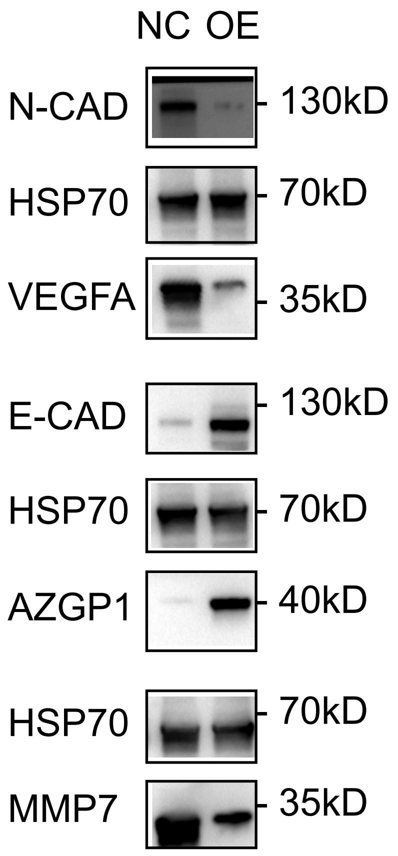

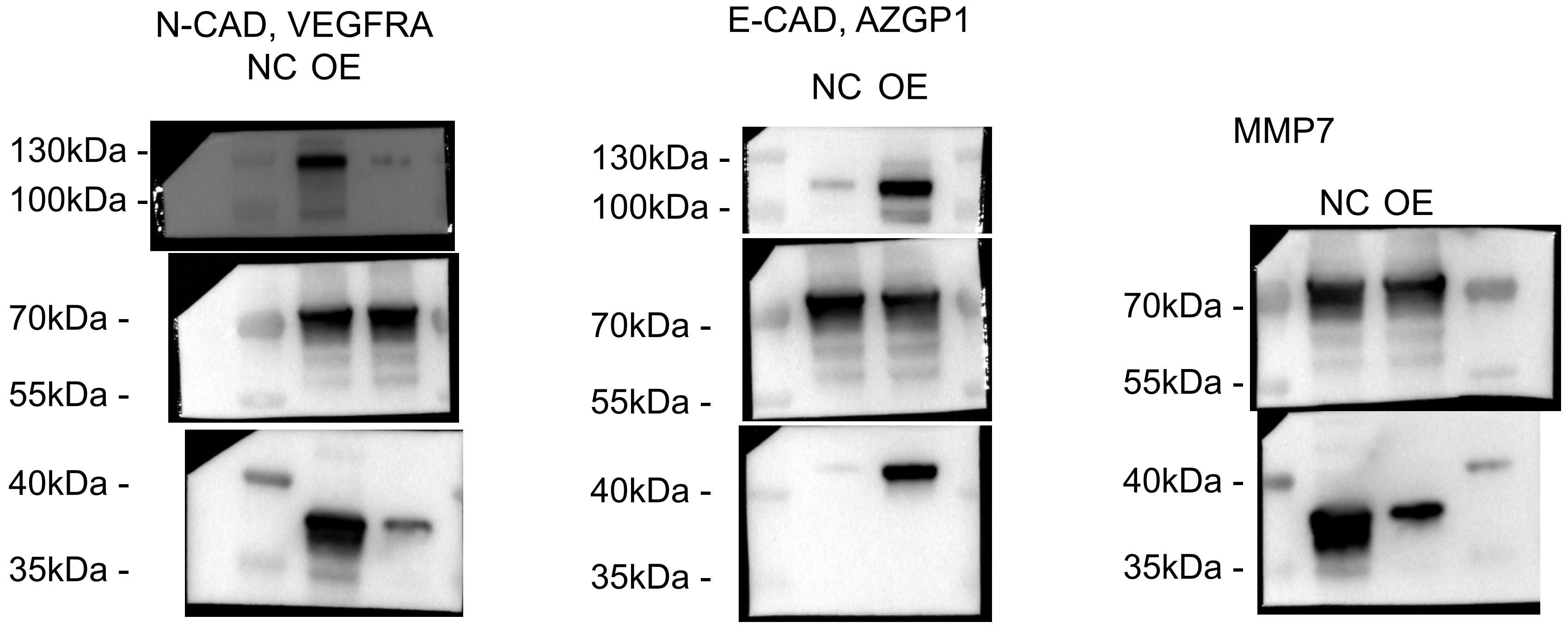

Combination

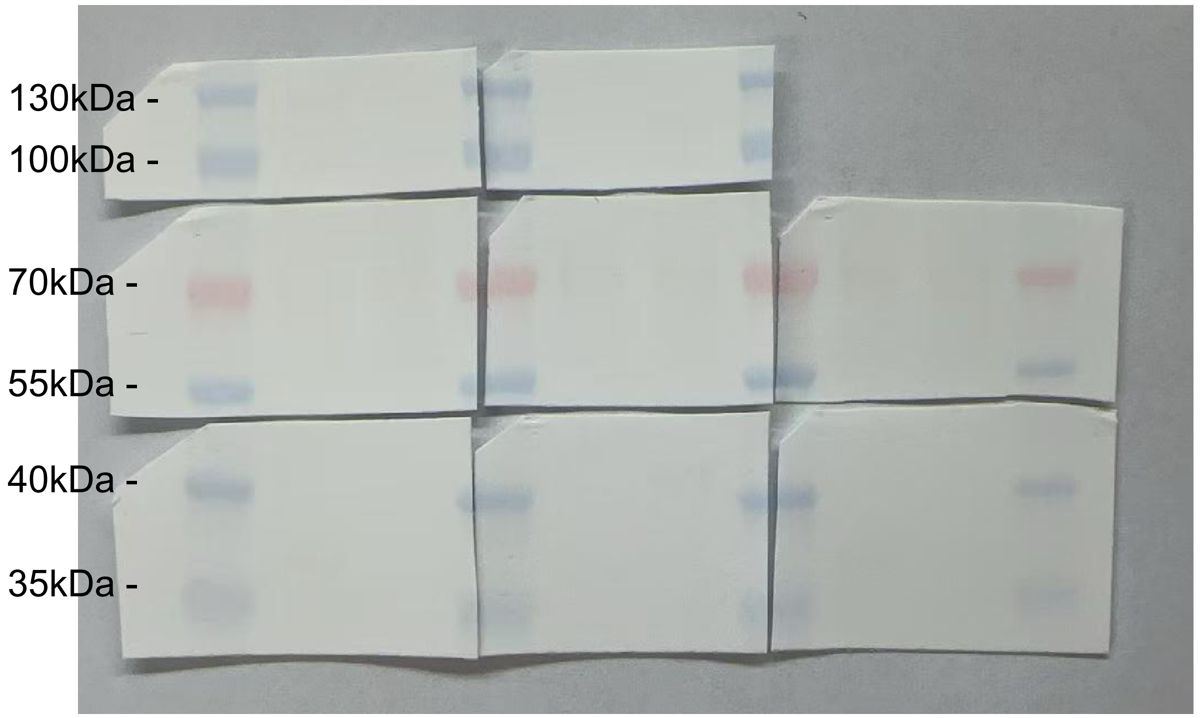

Supplementary Fig4

Raw western blots-OE rep3

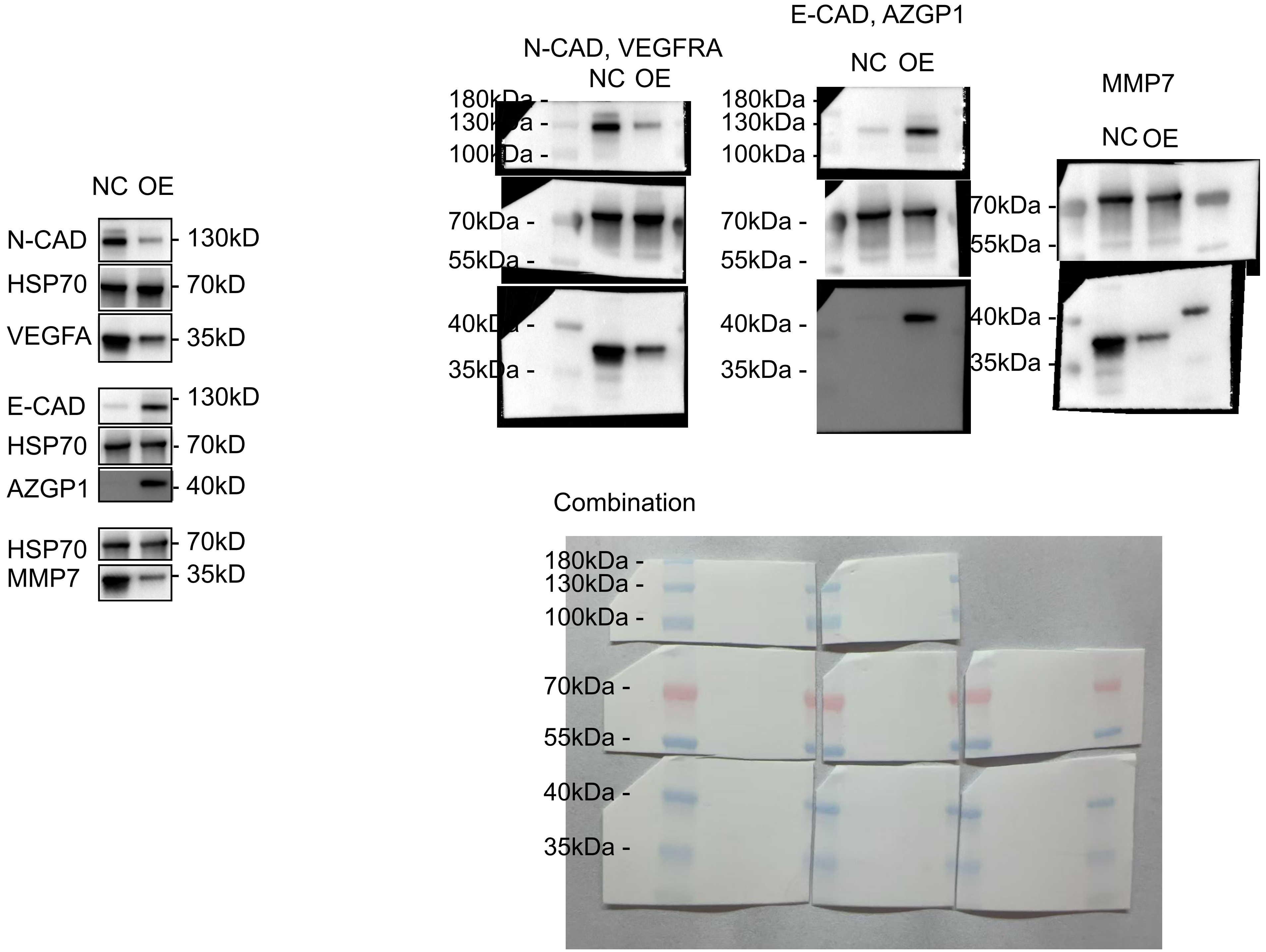

Supplementary Fig4

Raw western blots-SI rep1

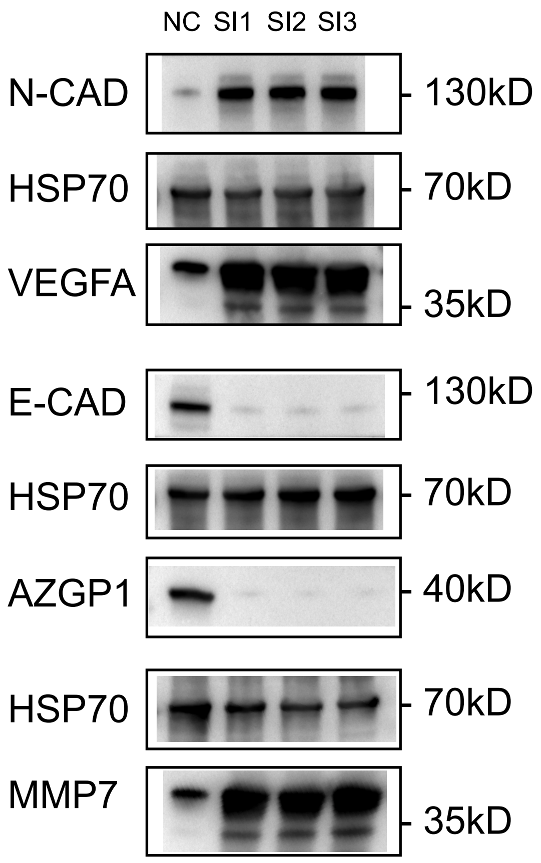

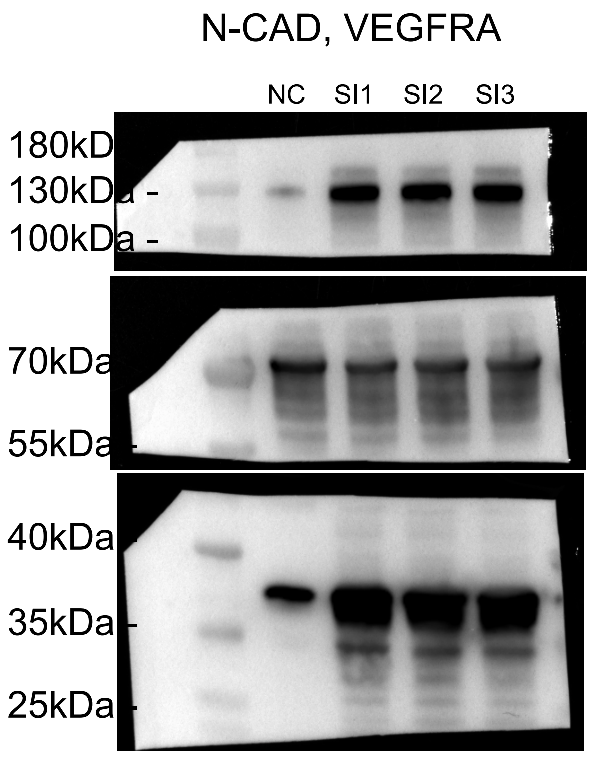

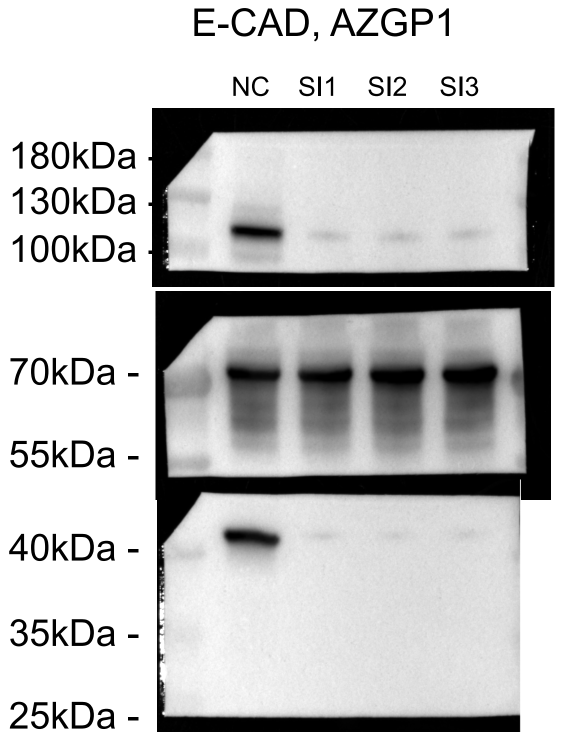

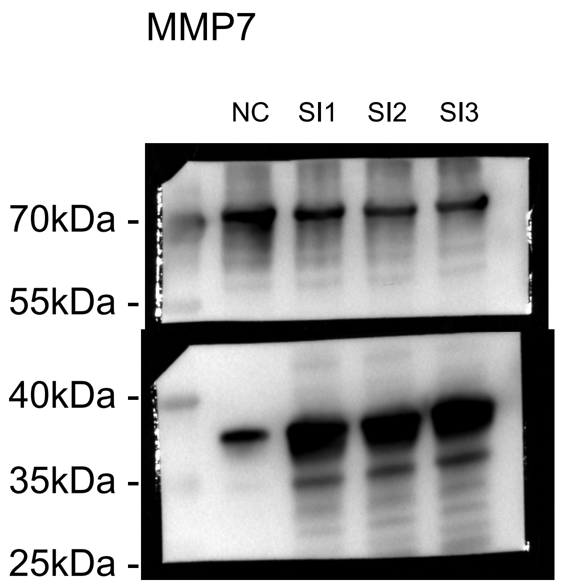

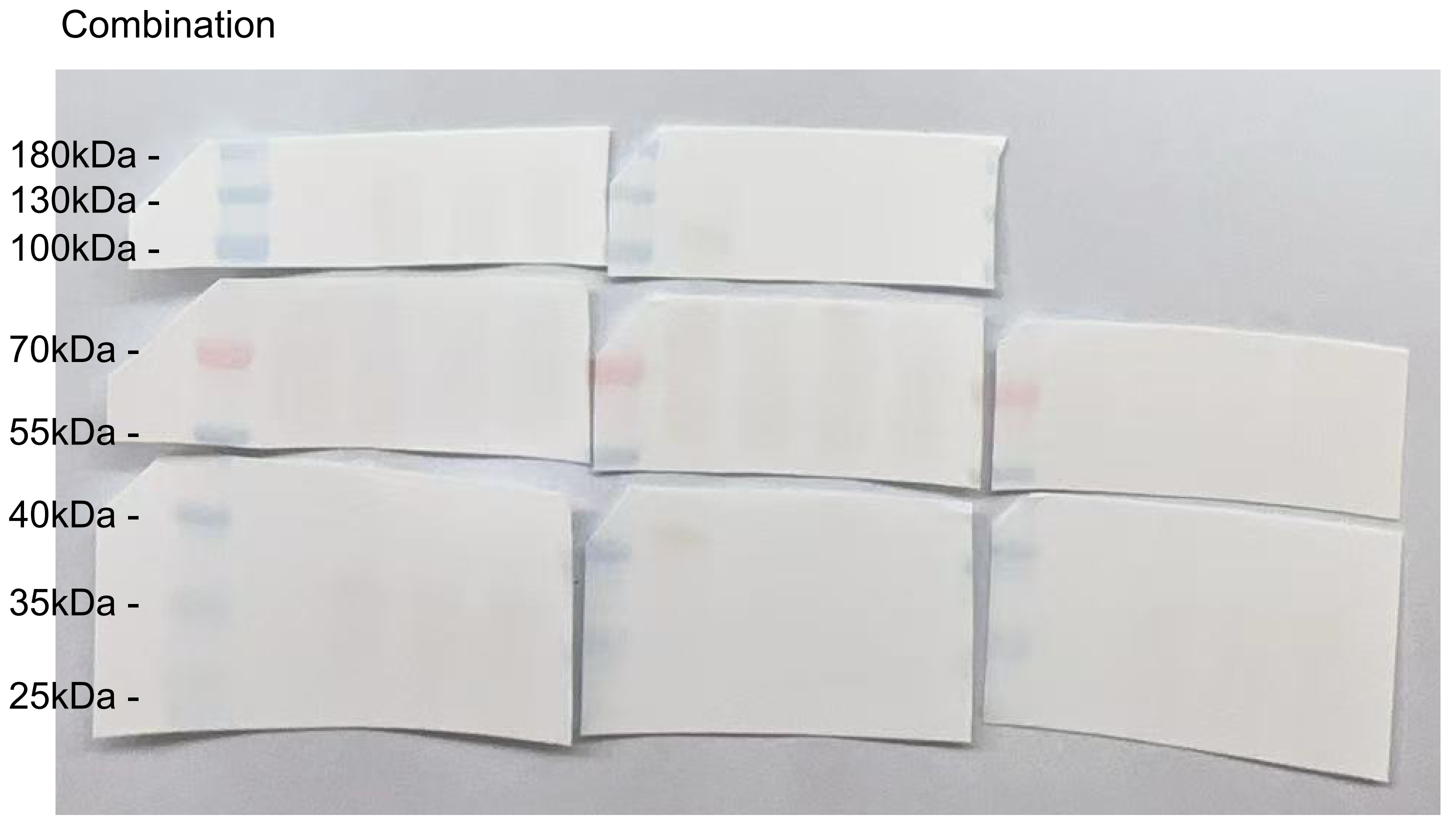

Supplementary Fig4

Raw western blots-SI rep2

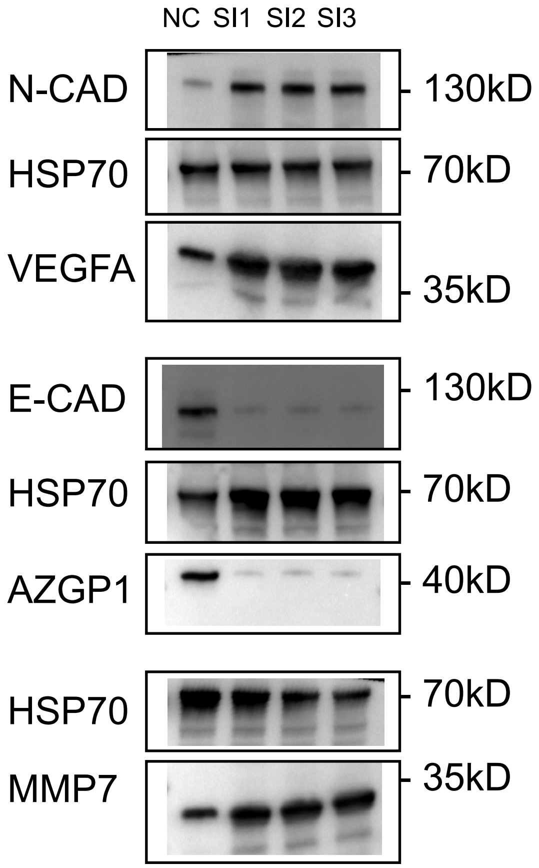

N-CAD, VEGFRA

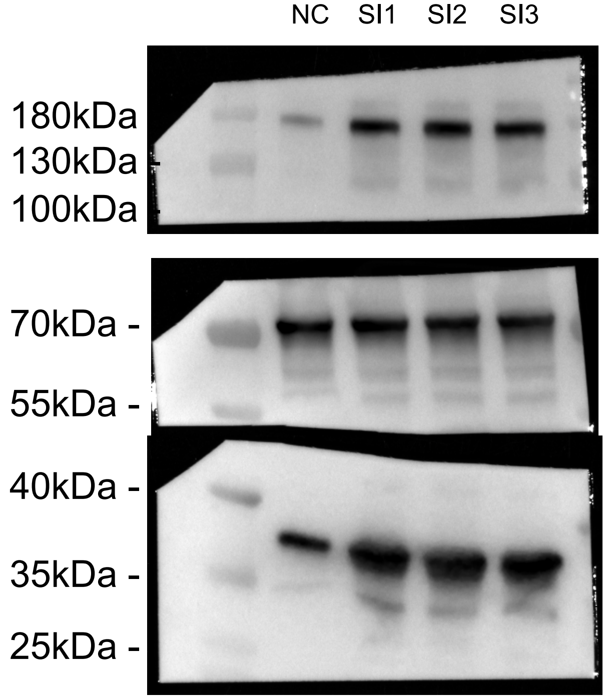

E-CAD, AZGP1

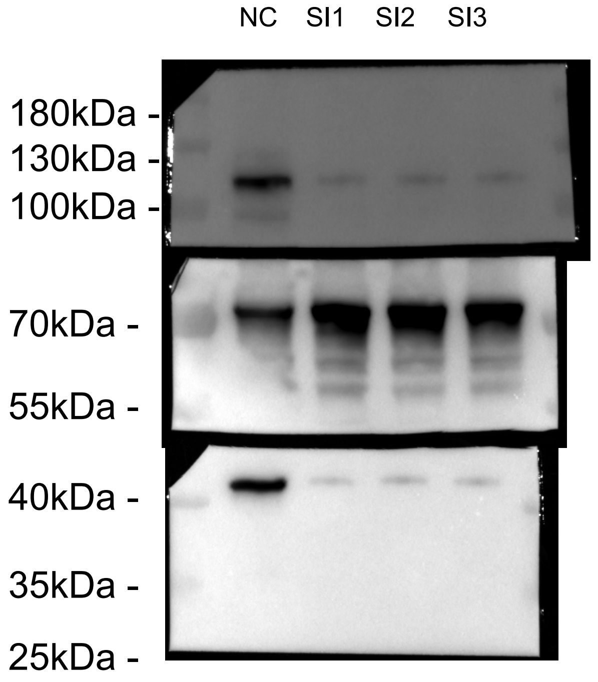

MMP7

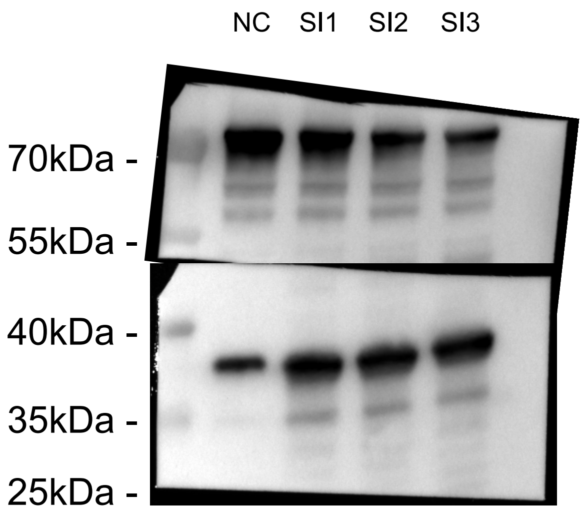

Combination

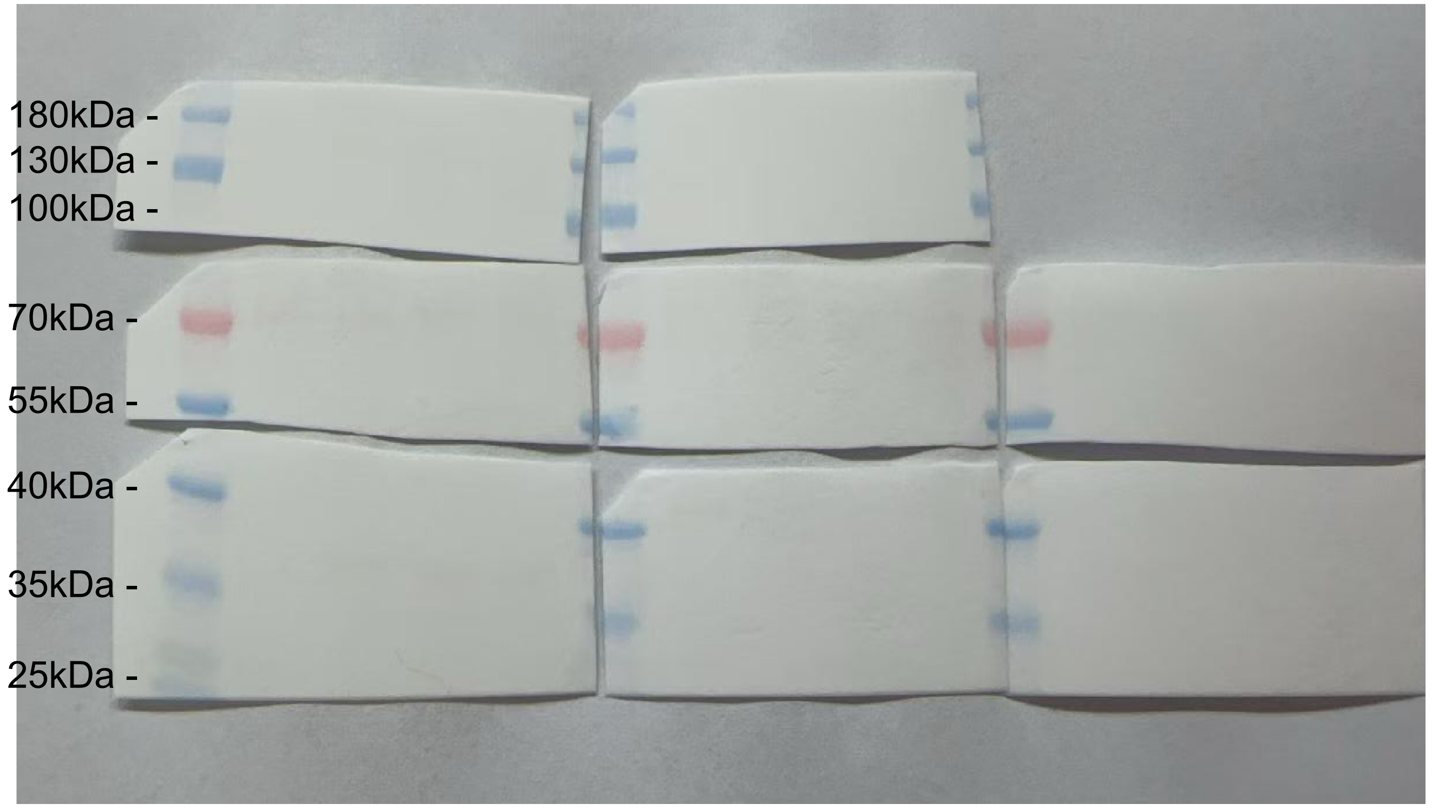

Supplementary Fig4

Raw western blots-SI rep3

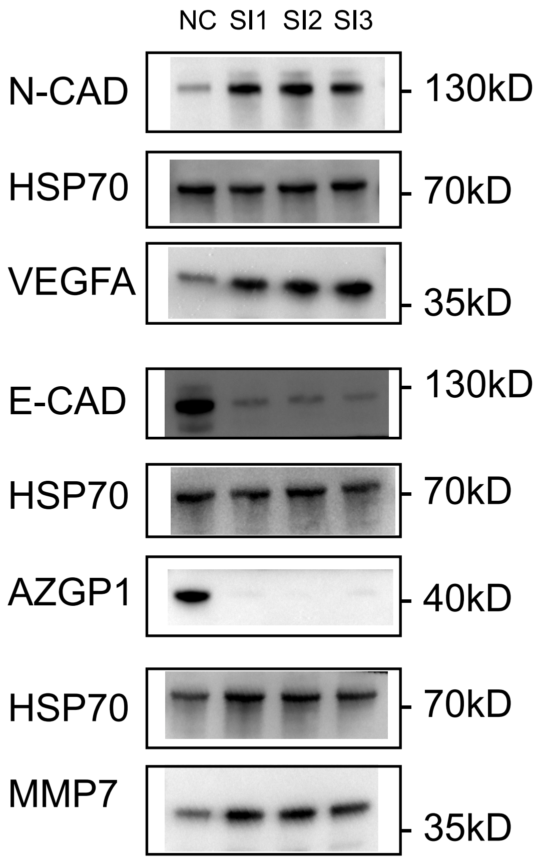

N-CAD, VEGFRA

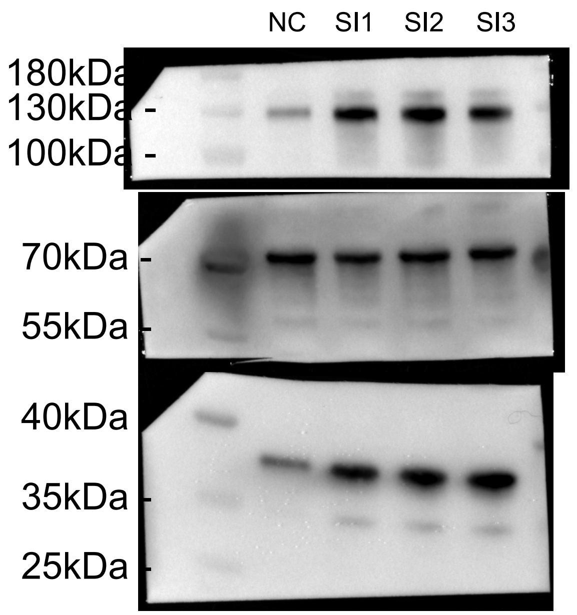

E-CAD, AZGP1

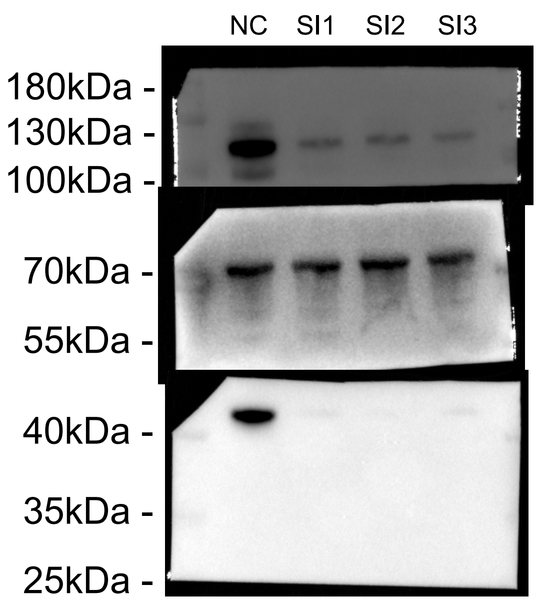

MMP7

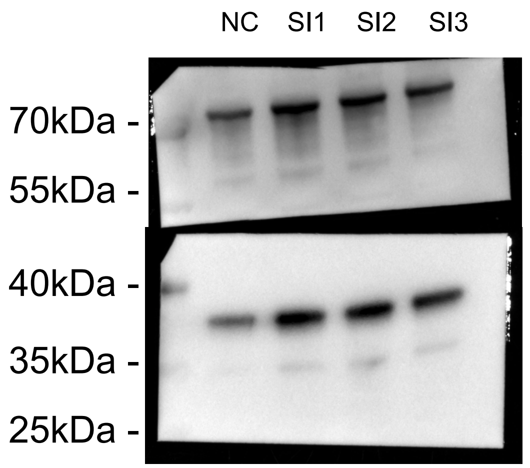

Combination

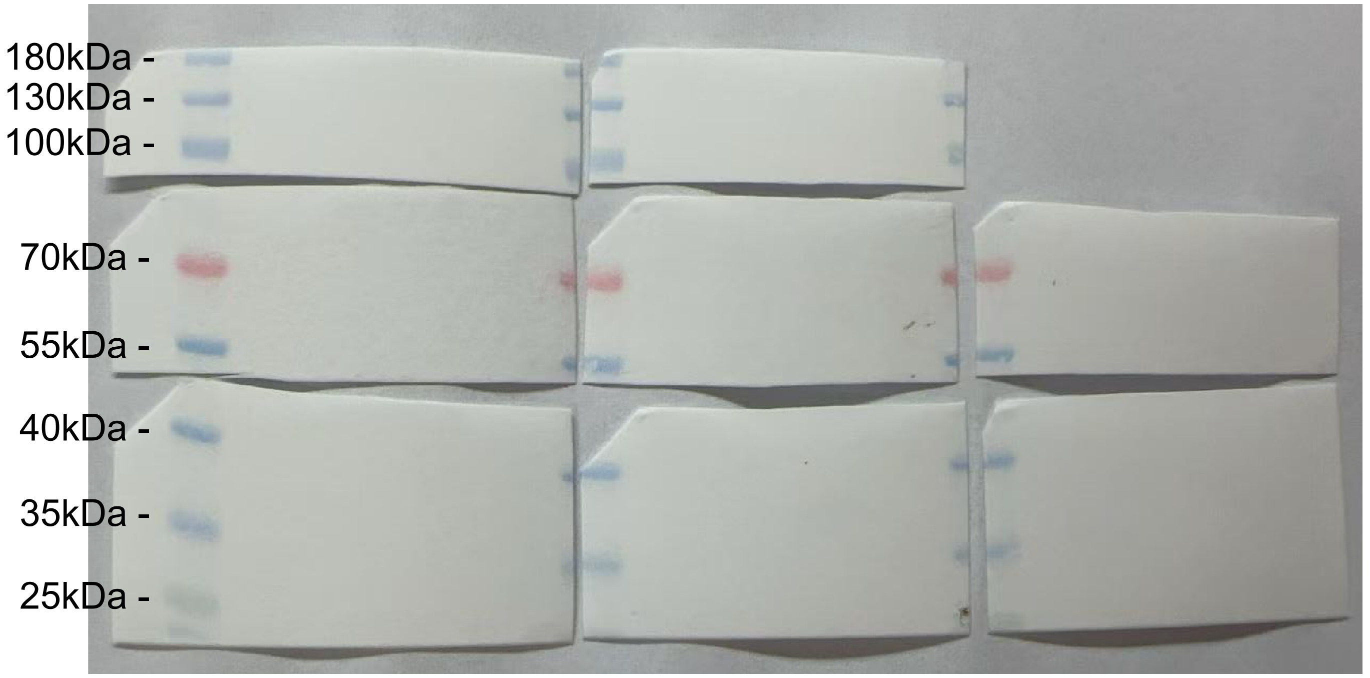

Supplement: Supplementary file 5 — Supplementary Material 5 [file 11658_2025_818_MOESM5_ESM.pdf]
